# From Exposure to Outcome: Air Pollution-Induced Oxidative Stress as a Determinant of Early and Late Outcomes After Coronary Artery Bypass Grafting

**DOI:** 10.3390/antiox15080930

**Published:** 2026-07-27

**Authors:** Tomasz Urbanowicz, Krzysztof J. Filipiak

**Affiliations:** 1 Cardiac Surgery and Transplantology Department, Poznan University of Medical Sciences, 61-848 Poznań, Poland; 2 The Center of Postgraduate Medical Education, 99/103 Marymoncka Street, 01-813 Warsaw, Poland

**Keywords:** coronary artery bypass grafting, air pollution, oxidative stress, environmental oxidative priming, particulate matter (PM2.5), endothelial dysfunction, mitochondrial dysfunction, cardiovascular exposome

## Abstract

Coronary artery bypass grafting (CABG) remains one of the most effective treatments for advanced coronary artery disease; however, substantial variability persists in both perioperative and long-term outcomes despite advances in surgical technique, myocardial protection, and risk stratification. Oxidative stress is a central mediator of tissue injury during cardiac surgery, contributing to ischemia–reperfusion injury, endothelial dysfunction, systemic inflammation, and postoperative organ complications. At the same time, chronic exposure to ambient air pollution has emerged as an important environmental determinant of cardiovascular disease through mechanisms that converge on many of the same redox-sensitive pathways. We propose the concept of environmental oxidative priming, whereby long-term exposure to particulate matter, nitrogen oxides, ozone, and other pollutants establishes a persistent state of endothelial dysfunction, mitochondrial impairment, chronic inflammation, nitric oxide depletion, and reduced antioxidant reserve before surgery. Within this framework, CABG represents a second oxidative challenge superimposed on a pre-existing environmentally conditioned phenotype. We discuss the mechanistic overlap between air pollution-induced cardiovascular injury and cardiac surgical stress and examine how this interaction may contribute to postoperative complications, graft adaptation, major adverse cardiovascular events, and long-term survival. Recognition of air pollution as a modifier of biological resilience provides a novel framework for understanding outcome heterogeneity after CABG and may support future precision-based risk stratification and preventive strategies.

## 1. Introduction

Coronary artery bypass grafting (CABG) remains a cornerstone of treatment for advanced coronary artery disease, providing durable symptom relief and survival benefit in appropriately selected patients [1,2,3]. Despite advances in operative technique, myocardial protection, perioperative care, and secondary prevention, substantial heterogeneity persists in both early and long-term outcomes [4,5]. Patients with apparently similar clinical and procedural profiles may experience markedly different trajectories, ranging from uncomplicated recovery and durable graft function to postoperative atrial fibrillation, neurological complications, graft failure, recurrent ischemic events, or premature death [6,7]. This variability indicates that clinically relevant determinants of perioperative resilience remain incompletely captured by current risk models.

Contemporary risk models, including EuroSCORE II and the Society of Thoracic Surgeons score, estimate perioperative risk primarily from demographic characteristics, comorbidities, cardiac function, and procedural complexity [8,9]. Environmental exposures are not incorporated, despite substantial evidence linking air pollution to cardiovascular morbidity and mortality [10,11]. Chronic exposure to particulate and gaseous pollutants has been associated with endothelial dysfunction, vascular inflammation, autonomic imbalance, thrombogenicity, and accelerated atherosclerosis [12,13,14]. Many of these effects are mediated through redox-sensitive pathways that are also central to the biological response to cardiac surgery [15,16].

Air pollution exerts systemic cardiovascular effects through interactions among pulmonary inflammation, circulating immune cells, platelets, vascular endothelium, smooth muscle cells, and mitochondria [10]. Repeated exposure may produce cumulative changes in vascular and metabolic homeostasis that remain clinically silent until the organism is challenged by a major physiological stressor. CABG therefore provides a clinically relevant setting in which the consequences of chronic environmental exposure may become apparent.

Air pollution and cardiac surgery converge on several biological processes, including reactive oxygen species generation, endothelial dysfunction, mitochondrial injury, nitric oxide depletion, inflammatory activation, thrombogenicity, and impaired tissue repair. These pathways influence organs commonly affected by postoperative complications, including the myocardium, brain, kidneys, and vascular conduits. Nevertheless, environmental cardiovascular research and cardiac surgical research have largely evolved in parallel, with limited integration of exposure assessment into perioperative risk evaluation.

The biological response to CABG is shaped not only by procedural factors but also by the cumulative effects of aging, genetics, comorbid disease, lifestyle, and environmental exposure. Long-term air pollution exposure may therefore represent an underrecognized modifier of perioperative susceptibility by altering vascular, mitochondrial, and inflammatory reserve before surgery [17,18,19,20].

In this review, we introduce the concept of environmental oxidative priming to describe a preoperative biological state created by chronic pollutant exposure and characterized by reduced redox, endothelial, mitochondrial, and inflammatory reserve. Within this framework, CABG acts as an acute oxidative challenge superimposed on a chronically conditioned host. We examine the mechanistic and clinical evidence supporting this model and consider how it may contribute to variability in postoperative complications, graft adaptation, recurrent cardiovascular events, and long-term survival.

This narrative review was developed following a structured literature search performed in PubMed/MEDLINE, Embase, Web of Science, and Scopus in July 2026. Searches were conducted using combinations of Medical Subject Headings (MeSH) and free-text terms including “coronary artery bypass grafting”, “air pollution”, “oxidative stress”, “particulate matter”, “PM2.5”, “nitrogen dioxide”, “ozone”, “endothelial dysfunction”, “mitochondrial dysfunction”, “ischemia-reperfusion injury”, and “cardiovascular outcomes”, combined using Boolean operators (AND/OR). Priority was given to original studies, systematic reviews, meta-analyses, and major guideline documents published in English. Because the objective of this article was to provide a mechanistic narrative synthesis rather than a systematic review, formal PRISMA screening procedures and quantitative evidence synthesis were not performed.

Qualitative descriptors used in the tables do not represent formal GRADE or Oxford CEBM classifications. They reflect the consistency, methodological robustness, reproducibility, and biological plausibility of the available evidence. No formal risk-of-bias assessment, inter-rater agreement analysis, or validated evidence-grading procedure was applied.

## 2. CABG as a Human Model of Acute Oxidative Injury

Among all cardiovascular interventions, CABG occupies a unique position as a highly reproducible model of acute oxidative and inflammatory stress [21,22,23]. Unlike spontaneous cardiovascular events, which occur under heterogeneous biological conditions, cardiac surgery exposes patients to a predictable sequence of physiological insults that simultaneously activate multiple oxidative pathways. Surgical trauma, extracorporeal circulation, ischemia–reperfusion injury, hemolysis, endothelial activation, and systemic inflammation collectively generate one of the most profound oxidative challenges encountered in clinical medicine [24].

Oxidative injury begins with the earliest stages of surgical tissue damage. Tissue injury activates innate immune responses and stimulates the release of inflammatory mediators that recruit neutrophils, monocytes, and platelets [25]. These cells generate reactive oxygen species as part of the host response to injury [26]. While such mechanisms are essential for tissue repair, excessive activation contributes to widespread oxidative stress and endothelial dysfunction.

The most significant oxidative burden, however, occurs during myocardial ischemia and subsequent reperfusion [27,28]. Temporary interruption of coronary blood flow results in accumulation of metabolic intermediates, alterations in mitochondrial respiration, intracellular calcium overload, and activation of multiple stress pathways [29]. Restoration of blood flow, although necessary for tissue survival, paradoxically produces an abrupt increase in reactive oxygen species generation [30]. This phenomenon, known as ischemia–reperfusion injury, represents a major source of myocardial and systemic oxidative stress during CABG.

Mitochondria constitute the principal intracellular source of reactive oxygen species during reperfusion [31,32]. During ischemia, disruption of oxidative phosphorylation alters electron transport chain function and promotes accumulation of reduced substrates [33,34]. Upon reperfusion, the sudden reintroduction of oxygen leads to excessive electron leakage and reactive oxygen species generation [35]. The resulting oxidative injury affects cellular membranes, proteins, nucleic acids, and intracellular signaling pathways [36]. In severe cases, these alterations contribute to mitochondrial permeability transition, apoptosis, and myocardial dysfunction [37].

Cardiopulmonary bypass further amplifies these processes. Exposure of circulating blood to artificial extracorporeal surfaces activates complement pathways, leukocytes, platelets, and coagulation systems [38,39]. Simultaneously, hemolysis releases free hemoglobin and catalytic iron, both of which contribute to oxidative reactions [40]. The consequence is a systemic inflammatory response characterized by endothelial activation, cytokine release, oxidative stress, and microvascular dysfunction [41,42]. Although advances in perfusion technology have reduced some of these effects, cardiopulmonary bypass remains a major generator of oxidative injury.

The consequences of oxidative stress extend beyond the myocardium. The kidneys, brain, lungs, and vascular system are all exposed to the inflammatory and oxidative environment generated during surgery [43,44]. Postoperative atrial fibrillation, acute kidney injury, neurological complications, impaired graft adaptation, and increased mortality have all been associated with excessive reactive oxygen species generation and endothelial dysfunction [45,46,47]. This observation highlights the systemic nature of oxidative injury during CABG and reinforces its relevance as a determinant of outcome.

Although the two-hit paradigm was originally developed to explain disease progression in fields such as hepatology and oncology, the conceptual framework has subsequently been adopted in cardiovascular biology to describe situations in which chronic subclinical injury modifies susceptibility to an acute pathological insult. In the present review, the model is used as a conceptual rather than disease-specific framework, illustrating how long-term environmentally induced oxidative stress may lower biological resilience before the acute oxidative challenge imposed by CABG.

**Figure 1:**
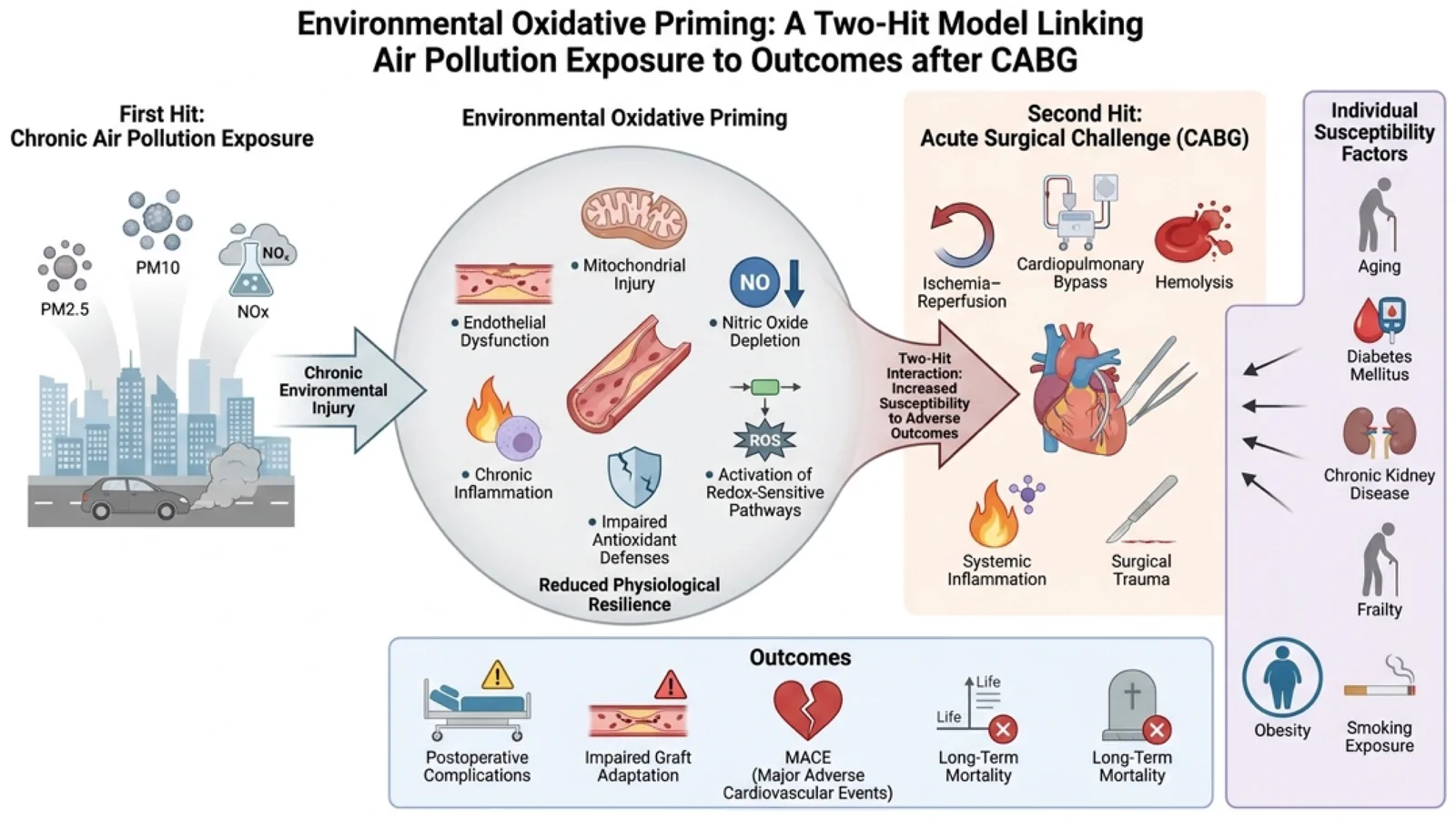
Environmental Oxidative Priming: A Two-Hit Model Linking Air Pollution Exposure to Outcomes after CABG. Created by www.figurelabs.ai (by Urbanowicz T, 2026). Arrow notation: Solid arrows (→) represent mechanistic progression or causal biological relationships. Converging arrows illustrate the cumulative effects of multiple pathways on postoperative outcomes. Abbreviations: CABG, coronary artery bypass grafting; MACE, major adverse cardiovascular events; NO, nitric oxide; PM, particulate matter; ROS, reactive oxygen species.

The relationship between air pollution and adverse cardiovascular outcomes has traditionally been examined from an epidemiological perspective [48,49]. However, the potential influence of environmental exposures on outcomes after coronary artery bypass grafting remains poorly conceptualized and limited to primary reports [50,51,52]. Figure 1 presents the conceptual framework underpinning this review and illustrates how chronic environmental exposure may modify the biological response to the acute oxidative stress associated with CABG.

The proposed model distinguishes two temporally distinct forms of oxidative stress. Chronic environmental exposure produces a sustained low-grade redox imbalance that progressively remodels vascular and mitochondrial function, whereas CABG induces an acute oxidative burst dominated by ischemia–reperfusion injury and cardiopulmonary bypass. Their sequential interaction forms the conceptual basis of environmental oxidative priming.

## 3. Environmental Oxidative Priming Before CABG: Determinants of Exposure

Air pollution exposure is heterogeneous and depends on geographic location, pollutant composition, and duration of exposure [53,54,55]. Urban environments are typically characterized by traffic- and industry-related pollutants, including PM2.5 (particulate matter with an aerodynamic diameter ≤ 2.5 μm), nitrogen oxides, and combustion-derived particles, whereas rural regions may exhibit greater contributions from agricultural activities, biomass combustion, and seasonal particulate emissions [56,57].

The biological effects of air pollution are influenced not only by pollutant concentration but also by pollutant composition and oxidative potential [58,59]. Traffic-derived particulate matter is particularly rich in transition metals and organic compounds that catalyze the generation of reactive oxygen species and promote endothelial injury [60,61]. Rural environments are characterized by agricultural emissions, biomass combustion, soil disturbance, and seasonal particulate generation [62].

From a CABG perspective, chronic exposure appears more relevant than short-term exposure. While acute pollution peaks may transiently affect endothelial function, autonomic balance, and thrombogenicity, long-term exposure promotes progressive endothelial dysfunction, mitochondrial injury, vascular remodeling, and depletion of antioxidant defenses [63,64]. These cumulative biological adaptations define the patient’s preoperative redox phenotype before surgical intervention. 

Seasonal variation provides an additional source of exposure heterogeneity. In many regions of the world, pollutant concentrations vary substantially with season, reflecting changes in atmospheric conditions, energy consumption, traffic patterns, and photochemical activity [65,66,67].

Seasonal fluctuations modify both the composition and biological effects of ambient air pollution. Winter months are generally associated with increased particulate matter resulting from domestic heating, fossil fuel combustion, and atmospheric inversions, whereas summer favors ozone formation through photochemical reactions. Although different pollutants predominate across seasons, both contribute to oxidative stress, endothelial dysfunction, and systemic inflammation [68,69,70,71]. Although different pollutants predominate across seasons, both contribute to oxidative stress, endothelial dysfunction, and systemic inflammation [72,73].

These seasonal differences may have clinical relevance because myocardial infarction, stroke, heart failure, and cardiovascular mortality also exhibit marked seasonal variation [74]. Although multiple environmental and behavioral factors contribute, seasonal fluctuations in pollution-related oxidative stress may represent one underrecognized determinant. Whether similar mechanisms influence perioperative vulnerability after CABG remains largely unexplored [75].

Although PM2.5 (Particulate Matter with an aerodynamic diameter ≤ 2.5 μm), PM10 (Particulate Matter with an aerodynamic diameter ≤ 10 μm), and nitrogen oxides are often analyzed collectively in epidemiological studies, they differ substantially in source, physicochemical behavior, biological targets, and mechanisms of cardiovascular injury. Appreciating these distinctions is essential because different pollutants may contribute to perioperative vulnerability through different pathways. Their principal exposure characteristics, dominant biological mechanisms, and potential relevance to CABG are summarized in Table 1.

### Pathophysiological Basis of Environmental Oxidative Priming

Environmental oxidative priming assumes that repeated exposure to particulate and gaseous pollutants induces persistent vascular, inflammatory, metabolic, and mitochondrial remodeling rather than transient physiological responses. Understanding the biological mechanisms linking inhaled pollutants to systemic cardiovascular injury is therefore fundamental to the proposed relationship between air pollution and CABG outcomes.

The lung serves as the primary interface between environmental exposure and the cardiovascular system [88]. Upon inhalation, airborne pollutants deposit throughout the respiratory tract according to their aerodynamic characteristics. Coarse particles such as PM10 predominantly accumulate within proximal airways, whereas fine and ultrafine particles penetrate deeply into the distal bronchioles and alveolar spaces [89]. Within these regions, pollutants interact directly with alveolar epithelial cells, pulmonary endothelial cells, and resident macrophages, initiating oxidative and inflammatory responses that extend far beyond the respiratory system.

Three complementary mechanisms appear to mediate the systemic cardiovascular effects of air pollution. The first involves direct translocation of ultrafine particles and particle-associated constituents across the alveolar-capillary barrier. Experimental studies have demonstrated that nanoparticles, transition metals, and combustion-derived organic compounds can enter the circulation, where they interact directly with vascular endothelium, circulating leukocytes, platelets, and distant organs. Although the quantitative contribution of particle translocation remains debated, even limited systemic dissemination may exert biologically significant effects through oxidative and inflammatory signaling pathways.

The second and perhaps most extensively characterized mechanism involves pulmonary inflammatory spillover. Exposure to particulate matter activates alveolar macrophages and airway epithelial cells, resulting in the release of pro-inflammatory cytokines, chemokines, and reactive oxygen species [90]. Mediators such as interleukin-1β, interleukin-6, tumor necrosis factor-α, and monocyte chemoattractant protein-1 subsequently enter the systemic circulation, promoting endothelial activation, leukocyte recruitment, and vascular inflammation [91]. In this model, the lung functions not merely as a target organ but as a biological amplifier that converts localized environmental injury into systemic cardiovascular stress.

A third pathway involves autonomic and neurogenic mechanisms [92,93]. Air pollutants stimulate pulmonary sensory receptors, including transient receptor potential ankyrin-1 channels and vagal afferent fibers, leading to alterations in autonomic balance characterized by sympathetic activation and reduced parasympathetic modulation. These changes contribute to endothelial dysfunction, increased vascular tone, blood pressure variability, and electrical instability within the myocardium. Such mechanisms may be particularly relevant to postoperative atrial fibrillation and other arrhythmias occurring after cardiac surgery.

Despite differing proximal mechanisms, these pathways converge on a common biological network characterized by oxidative stress, endothelial dysfunction, mitochondrial injury, nitric oxide depletion, and chronic low-grade inflammation. Progressive exhaustion of endogenous antioxidant defenses further reduces the capacity to withstand subsequent oxidative challenges, thereby establishing the persistent state of reduced biological resilience referred to here as environmental oxidative priming.

## 4. Distinct Pollutants, Distinct Biological Signatures

Ambient air pollution represents a complex mixture of particles and gases originating from multiple sources and possessing markedly different physicochemical properties [94,95]. These differences are not merely academic. They determine how pollutants interact with the cardiovascular system, influence oxidative stress pathways, and potentially affect outcomes following coronary artery bypass grafting.

Among the numerous environmental contaminants implicated in cardiovascular disease, fine particulate matter (PM2.5), coarse particulate matter (PM10), and nitrogen dioxide (NO_2_) have emerged as the most relevant candidates [96,97].

### 4.1. Airborne Particulate Matter with a Diameter of 2.5 μm or Less (PM2.5): The Dominant Cardiovascular Pollutant

Among all components of ambient air pollution, PM2.5 possesses the strongest and most consistent association with cardiovascular morbidity and mortality [98,99,100]. Its biological potency derives largely from its small aerodynamic diameter, which permits deep penetration into the distal airways and alveolar spaces [101]. Unlike larger particles, which are largely confined to the upper respiratory tract, PM2.5 reaches regions of the lung where inhaled air comes into intimate contact with the systemic circulation [102].

Yet particle size alone does not explain its toxicity. PM2.5 serves as a carrier for a diverse array of biologically active compounds, including transition metals, polycyclic aromatic hydrocarbons, sulfates, nitrates, and combustion-derived organic molecules. Many of these constituents possess substantial oxidative potential and directly participate in the generation of reactive oxygen species.

The cardiovascular effects of PM2.5 are increasingly recognized as manifestations of systemic vascular injury. Experimental studies have demonstrated impaired endothelium-dependent vasodilation within hours of exposure, accompanied by reduced nitric oxide bioavailability and increased expression of inflammatory mediators [103]. Beyond endothelial injury, PM2.5 disrupts mitochondrial homeostasis, promotes oxidative modification of lipoproteins, activates circulating immune cells, and alters autonomic regulation, thereby affecting multiple interconnected determinants of cardiovascular function.

These biological effects are particularly relevant to CABG because they compromise the systems responsible for perioperative resilience. Preserved endothelial integrity, mitochondrial function, and nitric oxide signaling are all essential for successful graft adaptation, tissue perfusion, and recovery from ischemia–reperfusion injury. Consequently, long-term PM2.5 exposure may alter the host response to surgery before the operative insult occurs.

Chronic exposure promotes vascular stiffness, endothelial senescence, and progressive depletion of physiological reserve. Consequently, patients exposed to high levels of PM2.5 may present for surgery with a cardiovascular system that is biologically older than would be predicted by chronological age alone.

### 4.2. Airborne Particulate Matter with Diameter of 10 μm or Less (PM10): Beyond the Lung

Historically, PM10 has often been regarded as less relevant to cardiovascular disease than PM2.5. This perception largely reflects differences in particle deposition. Because coarse particles are more likely to deposit within proximal airways, they were traditionally viewed as respiratory rather than cardiovascular pollutants [104].

Emerging evidence suggests that such distinctions are overly simplistic. Although PM10 may exert fewer direct vascular effects than PM2.5, it remains a potent stimulus for pulmonary inflammation [105]. Activation of alveolar macrophages and airway epithelial cells results in the release of cytokines, chemokines, and inflammatory mediators that subsequently enter the systemic circulation [106].

In this context, the lung functions not merely as a target organ but as a biological amplifier. Local inflammatory responses initiated by PM10 exposure become translated into systemic vascular effects through cytokine signaling, leukocyte activation, and oxidative stress pathways. The consequence is a state of chronic low-grade inflammation remarkably similar to that observed in patients with obesity, diabetes, and chronic kidney disease.

This distinction may have important implications for CABG. Whereas PM2.5 appears particularly relevant to endothelial and mitochondrial injury, PM10 may contribute predominantly through amplification of inflammatory responses. Such mechanisms could be especially important during the perioperative period when inflammatory activation already plays a central role in postoperative complications.

### 4.3. Nitrogen Dioxide (NO_2_): More than a Marker of Traffic Exposure

Nitrogen dioxide (NO_2_) occupies a unique position in cardiovascular environmental research [79,84]. Although traditionally regarded as a surrogate marker of traffic-related pollution, accumulating evidence indicates that NO_2_ itself contributes directly to vascular injury through mechanisms extending beyond its role as an indicator of complex urban pollutant mixtures [107,108]. 

The most significant of these may involve disruption of nitric oxide homeostasis. Endothelial health depends upon a delicate balance between nitric oxide production and oxidative degradation [109,110]. Chronic exposure to NO_2_ appears capable of shifting this balance toward oxidative consumption of nitric oxide, resulting in impaired vasodilation, increased vascular stiffness, and enhanced platelet activation.

Unlike particulate matter, which primarily promotes oxidative and inflammatory injury, NO_2_ appears to target vascular homeostasis more directly by impairing nitric oxide signaling. Progressive disruption of this pathway may compromise conduit function, endothelial adaptation, and cardiovascular resilience during the perioperative period.

**Figure 2:**
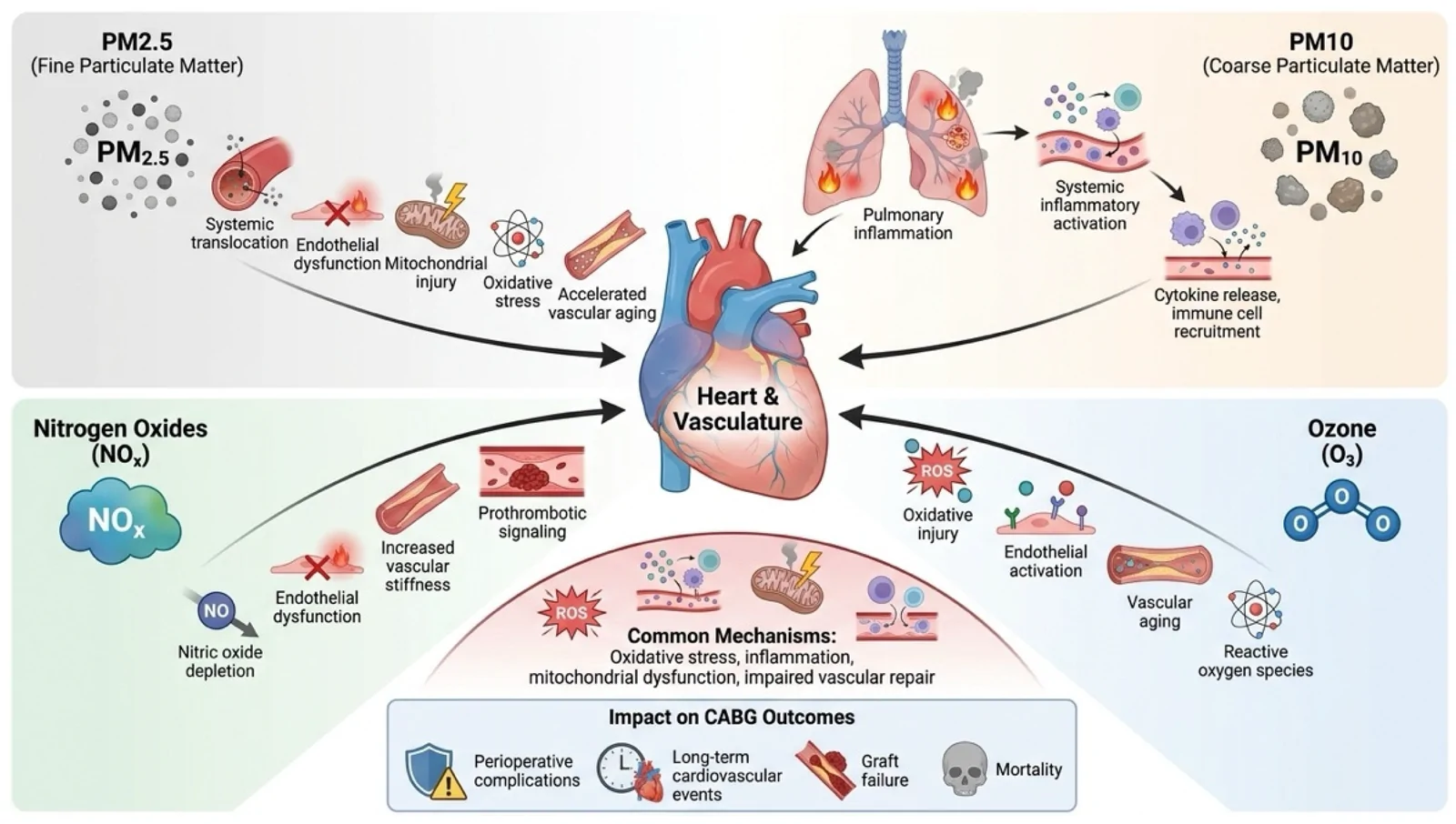
Distinct Biological Signatures of Major Air Pollutants Relevant to Coronary Artery Bypass Grafting. Created with www.figurelabs.ai (by Urbanowicz T, 2026). Arrow explanations: Solid arrows (→) represent causal or mechanistic relationships between pollutant exposure, shared pathogenic mechanisms, and CABG outcomes. Converging arrows indicate integration of multiple pollutant-specific pathways into common mechanisms of oxidative stress, inflammation, endothelial dysfunction, mitochondrial injury, and impaired vascular repair. The downward arrow denotes progression toward adverse perioperative and long-term clinical outcomes. Symbols indicating interruption (✕) represent inhibition or depletion of physiological protective mechanisms, particularly nitric oxide signaling and endothelial function. Abbreviations: CABG, coronary artery bypass grafting; NO, nitric oxide; NOx, nitrogen oxides; PM, particulate matter; ROS, reactive oxygen species.

Figure 2 summarizes the major pollutants relevant to CABG and highlights their predominant mechanistic pathways.

### 4.4. Pollutants as Biological Modifiers Rather than Isolated Risk Factors

Although PM2.5, PM10, and NO_2_ differ in their origins, deposition, and primary biological targets, they ultimately converge on a limited set of interconnected pathogenic pathways, including oxidative stress, endothelial dysfunction, mitochondrial injury, inflammation, and impaired vascular repair. Rather than acting as isolated cardiovascular risk factors, these pollutants should therefore be viewed as biological modifiers that shape the host response to surgical stress. Their overall impact is likely determined not only by individual exposure but also by interactions with aging, cardiovascular comorbidities, and the cumulative oxidative burden preceding CABG.

## 5. Converging Biological Pathways Between Air Pollution and CABG: The Mechanistic Basis of Environmental Oxidative Priming

**Figure 3:**
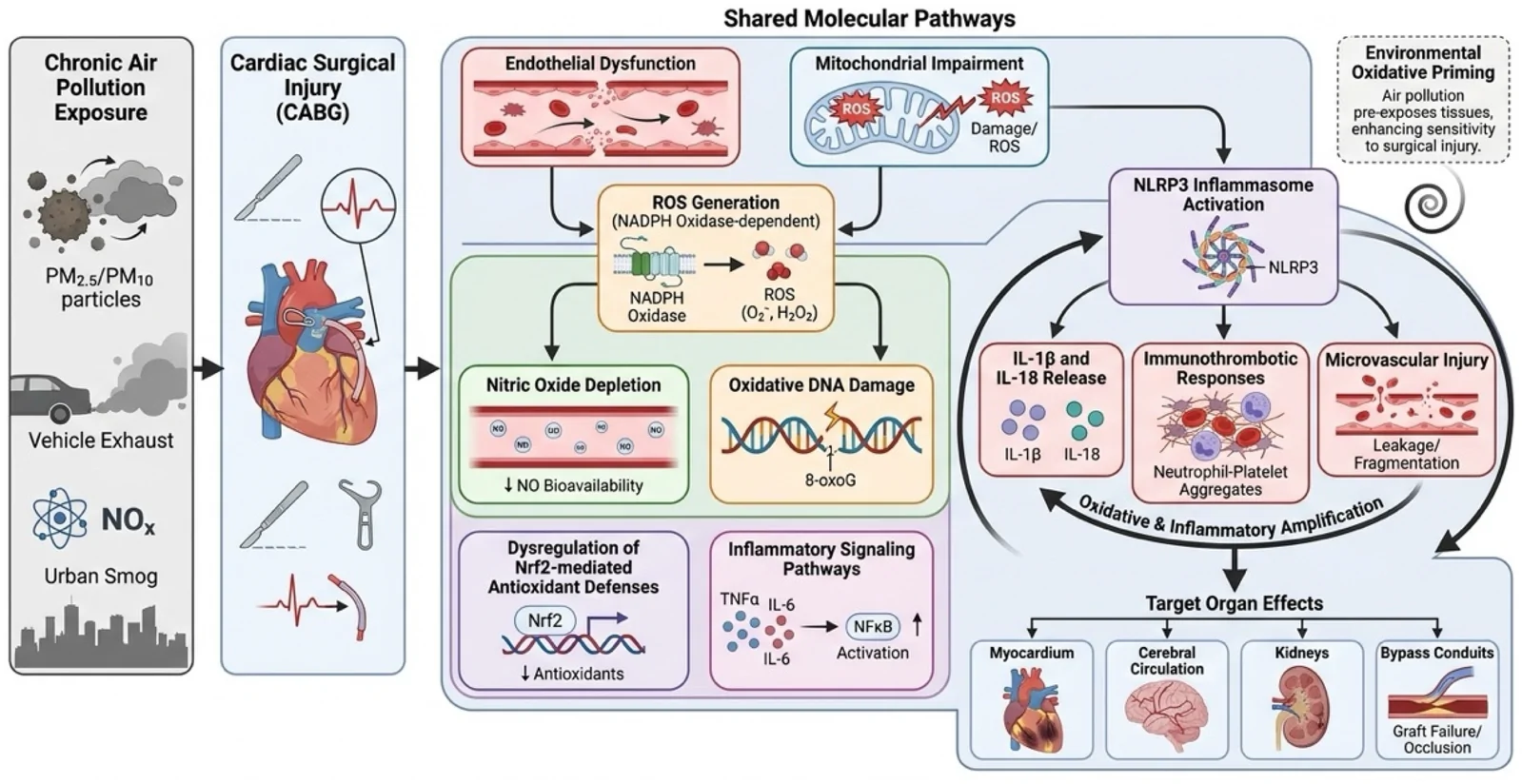
Shared Molecular Pathways Linking Air Pollution Exposure and Coronary Artery Bypass Grafting. Created with www.figurelabs.ai (by Urbanowicz T, 2026). Arrow explanations: Solid arrows (→) represent mechanistic progression between environmental exposure, surgical injury, and shared molecular pathways. Curved arrows indicate downstream signaling and biological amplification. Converging arrows illustrate the integration of multiple pathogenic mechanisms. Downward arrows (↓) indicate impaired physiological defense systems, including nitric oxide bioavailability and Nrf2-dependent antioxidant capacity. The circular pathway represents oxidative-inflammatory feed-forward amplification leading to target organ injury and adverse postoperative outcomes. Abbreviations: IL, interleukin; NLRP3, NOD-like receptor family pyrin domain-containing 3; NO, nitric oxide; Nrf2, nuclear factor erythroid 2-related factor 2; ROS, reactive oxygen species.

The central premise of this review is that chronic air pollution exposure and cardiac surgery converge on a limited set of interconnected biological pathways that govern oxidative stress, endothelial integrity, mitochondrial homeostasis, and inflammatory activation [111,112]. These shared mechanisms provide the biological framework for environmental oxidative priming (Figure 3).

### 5.1. Endothelial Dysfunction: The Earliest Point of Convergence

The vascular endothelium is the principal interface linking environmental exposure with cardiovascular homeostasis. Beyond regulating vascular tone, it coordinates inflammation, thrombosis, permeability, and tissue repair [113]. Consequently, even subtle endothelial dysfunction may profoundly influence perioperative resilience.

Experimental and human studies consistently demonstrate impaired endothelial-dependent vasodilation following exposure to PM2.5 and traffic-related pollutants. These changes are accompanied by increased oxidative stress, reduced nitric oxide bioavailability, enhanced expression of adhesion molecules, and recruitment of inflammatory cells into the vascular wall [114]. Over time, repeated exposure promotes chronic endothelial activation, accelerating atherosclerosis and impairing vascular resilience.

A strikingly similar pattern is observed during CABG. During CABG, cardiopulmonary bypass and ischemia–reperfusion injury similarly disrupt endothelial homeostasis, promoting glycocalyx degradation, leukocyte adhesion, microvascular dysfunction, and increased vascular permeability [115].

Importantly, endothelial dysfunction is not merely a marker of vascular disease; it determines the ability of tissues to adapt to physiological stress. Successful graft remodeling, myocardial perfusion, renal autoregulation, and cerebral blood flow all depend upon preserved endothelial function. Patients entering surgery with pre-existing pollution-induced endothelial injury may therefore possess diminished physiological reserve before the operative insult even begins.

### 5.2. Mitochondrial Dysfunction: The Central Hub of Oxidative Injury

Mitochondria are central regulators of cellular redox homeostasis and therefore occupy a pivotal position in the biological interface between chronic pollution exposure and surgical injury.

Chronic exposure to particulate matter disrupts mitochondrial respiration across multiple tissues. Experimental models have demonstrated reduced oxidative phosphorylation efficiency, impaired electron transport chain activity, altered mitochondrial dynamics, and increased mitochondrial ROS generation following pollutant exposure [116,117]. These abnormalities are accompanied by impaired ATP production, activation of inflammatory pathways, and progressive cellular dysfunction.

The same organelles become central mediators of injury during cardiac surgery. Ischemia–reperfusion injury triggers abrupt alterations in mitochondrial membrane potential, calcium homeostasis, and respiratory chain function [118]. The resulting burst of mitochondrial ROS generation contributes directly to myocardial stunning, apoptosis, endothelial injury, and systemic inflammation.

Air pollution may gradually impair mitochondrial reserve over years, while CABG imposes an acute metabolic challenge requiring optimal mitochondrial performance. Clinical outcomes may therefore depend not only on the magnitude of surgical stress but also on the pre-existing health of the mitochondrial network.

### 5.3. Nitric Oxide Biology and Loss of Vascular Homeostasis

Nitric oxide is a key regulator of vascular homeostasis, controlling vascular tone, platelet activation, inflammatory signaling, and endothelial integrity. Preservation of this pathway is therefore essential for both cardiovascular health and recovery after CABG.

One of the most consistent consequences of exposure to air pollution is the disruption of nitric oxide homeostasis [119]. Reactive oxygen species generated after pollutant exposure rapidly react with nitric oxide to form peroxynitrite, thereby reducing nitric oxide bioavailability and promoting oxidative tissue injury. Simultaneously, chronic oxidative stress promotes uncoupling of endothelial nitric oxide synthase, transforming an enzyme designed to generate nitric oxide into a source of additional superoxide production.

This process is remarkably relevant to CABG. Reduced nitric oxide bioavailability contributes to impaired graft adaptation, microvascular dysfunction, increased platelet activation, and postoperative organ injury. Furthermore, arterial conduits such as the internal thoracic artery derive much of their long-term success from preserved nitric oxide signaling. Environmental factors that disrupt this pathway may therefore influence not only perioperative physiology but also long-term conduit biology.

### 5.4. NADPH Oxidase and Amplification of Oxidative Stress

NADPH oxidases are dedicated enzymatic sources of reactive oxygen species and play a central role in sustaining oxidative injury after both chronic pollution exposure and cardiac surgery. Activation of endothelial cells, vascular smooth muscle cells, macrophages, and circulating leukocytes results in persistent ROS production that extends well beyond the initiating stimulus [120].

NADPH oxidase activation is similarly observed following cardiac surgery, particularly during ischemia–reperfusion and inflammatory activation [121]. Importantly, ROS generated through NADPH oxidase pathways further impair mitochondrial function, creating a self-perpetuating cycle in which oxidative stress becomes increasingly difficult to contain.

This interaction highlights a recurring theme within environmental cardiovascular biology: the biological consequences of pollution often persist long after the exposure itself has ended. In this sense, oxidative stress is not merely an acute response but a pathological state that can modify subsequent responses to surgical injury.

### 5.5. Nrf2 Dysfunction: Failure of Endogenous Defense Mechanisms

Nuclear factor erythroid 2-related factor 2 (Nrf2) is the principal transcriptional regulator that coordinates endogenous antioxidant defense [122]. Activation of Nrf2 induces numerous cytoprotective enzymes, including superoxide dismutase, catalase, glutathione-related enzymes, and heme oxygenase-1, thereby preserving cellular redox homeostasis [123]. Under physiological conditions, this system provides an adaptive response to oxidative stress and helps preserve cellular homeostasis.

Chronic exposure to air pollution may overwhelm or dysregulate these protective pathways. Experimental studies suggest that prolonged exposure to particulate matter impairs Nrf2 signaling, thereby reducing antioxidant capacity and increasing susceptibility to oxidative injury. Similar disturbances have been reported following ischemia–reperfusion injury and cardiopulmonary bypass.

The implications are substantial. If environmental exposure progressively weakens endogenous antioxidant defenses, patients may enter surgery with diminished capacity to respond to the oxidative stress generated during CABG. This concept aligns closely with the notion of environmental oxidative priming and provides a mechanistic explanation for differences in postoperative resilience.

### 5.6. Inflammation as a Consequence Rather than a Parallel Process

Persistent reciprocal amplification between oxidative stress and inflammation may therefore represent one of the principal mechanisms through which chronic environmental exposure lowers the threshold for excessive postoperative tissue injury. Chronic air pollution establishes persistent low-grade inflammatory activation, whereas CABG induces an acute inflammatory burst. Their interaction may amplify postoperative tissue injury.

### 5.7. Environmental Oxidative Priming: A Unifying Mechanistic Framework

Collectively, the mechanisms discussed above define the biological framework of environmental oxidative priming, whereby chronic pollutant exposure progressively reduces physiological resilience before the acute oxidative challenge imposed by CABG. Their principal points of convergence are summarized in Table 2.

### 5.8. NLRP3 Inflammasome Activation, Immunothrombosis, and Amplification of Oxidative Injury

While oxidative stress and inflammation are frequently discussed as parallel consequences of environmental exposure and cardiac surgery, accumulating evidence suggests that both processes are closely integrated through activation of the innate immune system. Among the molecular platforms linking oxidative injury to inflammatory signaling, the NLRP3 inflammasome has emerged as one of the most important regulators of cardiovascular inflammation and tissue damage [133].

The NLRP3 inflammasome is a multiprotein intracellular complex activated in response to a wide range of danger signals, including reactive oxygen species, mitochondrial dysfunction, oxidized lipids, extracellular ATP, and cellular injury [134,135]. Following activation, NLRP3 promotes caspase-1–dependent maturation and release of the pro-inflammatory cytokines interleukin-1β and interleukin-18, thereby amplifying local and systemic inflammatory responses. Although initially characterized as part of host defense mechanisms, excessive or persistent NLRP3 activation is increasingly recognized as a central contributor to cardiovascular disease.

Air pollution appears capable of activating this pathway through several complementary mechanisms [136]. Particulate matter induces mitochondrial dysfunction, increases intracellular reactive oxygen species generation, promotes the release of mitochondrial DNA, and enhances oxidative modification of lipids and proteins. These alterations act as potent danger-associated molecular patterns that stimulate inflammasome assembly within macrophages, endothelial cells, and vascular smooth muscle cells. Experimental studies have demonstrated increased NLRP3 activity following PM2.5 exposure, accompanied by endothelial dysfunction, vascular inflammation, accelerated atherosclerosis, and impaired nitric oxide signaling.

These mechanisms are highly relevant to CABG because postoperative atrial fibrillation, acute kidney injury, neurological injury, graft thrombosis, and microvascular dysfunction all involve coordinated inflammatory and thrombotic activation [41,137]. Environmental inflammasome priming may therefore lower the threshold for exaggerated postoperative immune responses.

Beyond its inflammatory effects, inflammasome activation also contributes to the development of a prothrombotic phenotype. The traditional distinction between inflammation and thrombosis has increasingly been replaced by the concept of immunothrombosis, whereby innate immune activation directly influences coagulation pathways, platelet function, and microvascular integrity. Activation of NLRP3 promotes expression of tissue factor, enhances platelet reactivity, increases endothelial permeability, and stimulates recruitment of neutrophils capable of releasing neutrophil extracellular traps (NETs) [138]. These highly thrombogenic structures provide a scaffold for platelet aggregation and fibrin deposition, thereby linking inflammatory activation to vascular occlusion.

The relevance of these mechanisms to CABG may be substantial. Postoperative complications such as atrial fibrillation, acute kidney injury, neurological dysfunction, microvascular injury, and graft thrombosis all involve varying degrees of inflammatory and thrombotic activation. Air pollution-induced inflammasome priming may therefore increase susceptibility to these complications by lowering the threshold for excessive postoperative inflammatory responses. In this context, chronic environmental exposure may not simply increase baseline oxidative stress but may fundamentally alter the intensity and duration of innate immune activation following surgery.

An additional implication concerns long-term outcomes after revascularization. Persistent low-grade activation of inflammasome signaling has been implicated in endothelial senescence, vascular remodeling, progression of atherosclerosis, and heart failure development [139,140]. These processes closely parallel the concept of persistent oxidative pressure proposed later in this review. Although accumulating mechanistic evidence supports this concept, prospective clinical studies are required to determine the extent to which environmental oxidative priming contributes to graft adaptation and long-term survival after CABG.

Overall, NLRP3 signaling represents a mechanistic bridge linking oxidative stress, endothelial dysfunction, innate immunity, and thrombosis. Future studies should determine whether inflammasome-related biomarkers improve perioperative risk stratification and identify patients most likely to benefit from targeted redox or immunomodulatory interventions.

The complexity of environmental oxidative priming suggests that future biological characterization will require multimarker approaches rather than reliance on individual circulating indicators. Candidate biomarkers representing the principal mechanistic domains discussed in this review are summarized in Table 3.

## 6. On-Pump Versus Off-Pump CABG as a Model of Environmental Oxidative Susceptibility

On-pump and off-pump CABG provide distinct models of oxidative stress exposure. Cardiopulmonary bypass induces a coordinated oxidative and inflammatory response characterized by ischemia–reperfusion injury, complement activation, endothelial dysfunction, leukocyte activation, and excessive generation of reactive oxygen species.

The concept of environmental oxidative priming suggests that chronic exposure to air pollution may modify the biological response to surgical insults. Patients with long-term exposure to PM2.5, PM10, or nitrogen oxides may enter surgery with pre-existing endothelial dysfunction, impaired mitochondrial function, and reduced antioxidant reserve. Consequently, an identical operative insult may elicit substantially different biological responses depending on the patient’s pre-existing environmental redox phenotype.

Viewed from this perspective, on-pump and off-pump CABG represent complementary biological models for investigating environmental oxidative susceptibility. Because the two techniques differ primarily in the magnitude of oxidative and inflammatory activation, they provide a unique opportunity to examine whether chronic exposure to pollution modifies the host response to varying levels of perioperative oxidative stress.

Rather than representing competing surgical strategies alone, on-pump and off-pump CABG may therefore serve as complementary experimental models for studying interactions between chronic environmental injury and acute surgical oxidative stress. Patient populations in whom these interactions are expected to be most pronounced are summarized in Table 4.

Off-pump CABG provides an informative contrast. By avoiding extracorporeal circulation and reducing the magnitude of ischemia–reperfusion injury, off-pump procedures substantially attenuate activation of oxidative and inflammatory pathways. Numerous studies have demonstrated lower concentrations of inflammatory mediators, reduced release of oxidative stress biomarkers, and diminished endothelial injury following off-pump surgery. While the clinical implications of these differences remain debated, the biological distinction is undeniable.

While the mechanistic overlap between pollution exposure and cardiac surgical injury is increasingly compelling, the strength of evidence linking environmental factors to specific cardiovascular outcomes remains heterogeneous. Some associations are supported by extensive epidemiological and experimental literature, whereas others remain largely hypothetical in surgical populations. Distinguishing established observations from emerging concepts is therefore important for interpreting the current state of the field. Table 5 summarizes the evidence base relevant to outcomes commonly encountered after CABG.

If environmental oxidative priming truly influences perioperative resilience, one would expect its biological impact to become most evident when the operative oxidative burden approaches the limits of the patient’s adaptive capacity. In this context, differences between on-pump and off-pump CABG may provide a clinically relevant model for exploring threshold effects, whereby patients with substantial environmental exposure derive greater benefit from strategies that minimize additional oxidative and inflammatory stress. Although this concept remains hypothetical, it provides a testable framework for future prospective studies that integrate environmental exposure assessment with surgical decision-making.

## 7. Susceptible Populations and Environmental Oxidative Priming

The biological effects of air pollution are unlikely to be uniform across all CABG patients. Instead, the biological consequences of environmental oxidative priming are likely to depend on the patient’s pre-existing capacity to maintain redox homeostasis, endothelial integrity, mitochondrial function, and adaptive inflammatory responses [184].

Diabetes mellitus represents one of the clearest clinical models of reduced biological resilience [185]. Chronic hyperglycemia, advanced glycation, endothelial dysfunction, mitochondrial impairment, and persistent inflammation establish a pre-existing oxidative phenotype that closely resembles the biological effects of long-term pollutant exposure [186]. This interaction may be particularly important for saphenous vein graft disease [187]. Diabetes promotes intimal hyperplasia, smooth muscle proliferation, endothelial dysfunction, and accelerated atherosclerosis within vein grafts. Air pollution may intensify these same processes by increasing oxidized LDL, vascular adhesion molecule expression, platelet activation, and inflammatory cell recruitment. Rather than acting independently, environmental and metabolic stressors are therefore likely to reinforce one another, further compromising tolerance to ischemia–reperfusion injury and cardiopulmonary bypass.

Chronic obstructive pulmonary disease and obesity may further amplify susceptibility through persistent inflammatory activation and systemic oxidative stress. Frailty and advanced age are also characterized by reduced physiological resilience, mitochondrial dysfunction, and impaired adaptive responses, potentially lowering the threshold for postoperative complications. Additional modifiers include smoking, socioeconomic disadvantage, and sex-specific differences in exposure patterns and vascular biology. These factors may influence both cumulative pollutant burden and biological responses to oxidative stress.

Chronic kidney disease is another phenotype in which pollution-induced oxidative stress may carry disproportionate consequences. Progressive renal dysfunction is accompanied by chronic inflammation, endothelial dysfunction, accumulation of uremic toxins, impaired nitric oxide signaling, and loss of antioxidant capacity, creating a state of markedly reduced physiological resilience before surgery. 

Sex-related differences in environmental susceptibility extend beyond simple epidemiological comparisons and reflect fundamental differences in vascular biology, immune regulation, oxidative signaling, and endothelial function. Postmenopausal women may enter surgery with reduced hormonal protection, smaller coronary vessels, greater microvascular disease, and higher inflammatory burden. Air pollution-induced endothelial dysfunction may therefore have different consequences in women, particularly for microvascular perfusion, postoperative heart failure, atrial fibrillation, and neurological injury.

Several additional clinical phenotypes are characterized by chronic oxidative and inflammatory activation before surgery. Chronic obstructive pulmonary disease, obesity, metabolic syndrome, and frailty each contribute to sustained redox imbalance, endothelial dysfunction, and impaired adaptive responses, thereby lowering the threshold for perioperative organ injury.

Smoking should not be regarded solely as a confounding variable but rather as a parallel environmental exposure that shares many mechanistic features with ambient air pollution [188]. Tobacco smoke and urban particulate pollution both contain oxidant gases, combustion-derived particles, transition metals, and organic compounds that promote endothelial dysfunction, oxidative injury, platelet activation, and vascular inflammation. Their combined effects are therefore likely to be synergistic rather than simply additive.

Biological aging further amplifies these processes through endothelial senescence, mitochondrial dysfunction, reduced antioxidant capacity, and impaired regenerative potential. Consequently, chronological age may only partially capture the decline in physiological resilience relevant to cardiac surgery. Frailty may be a more informative phenotype because it reflects reduced adaptive capacity across multiple systems. Frail patients frequently demonstrate inflammation, sarcopenia, mitochondrial dysfunction, impaired endothelial repair, reduced antioxidant capacity, and diminished physiological reserve. 

**Figure 4:**
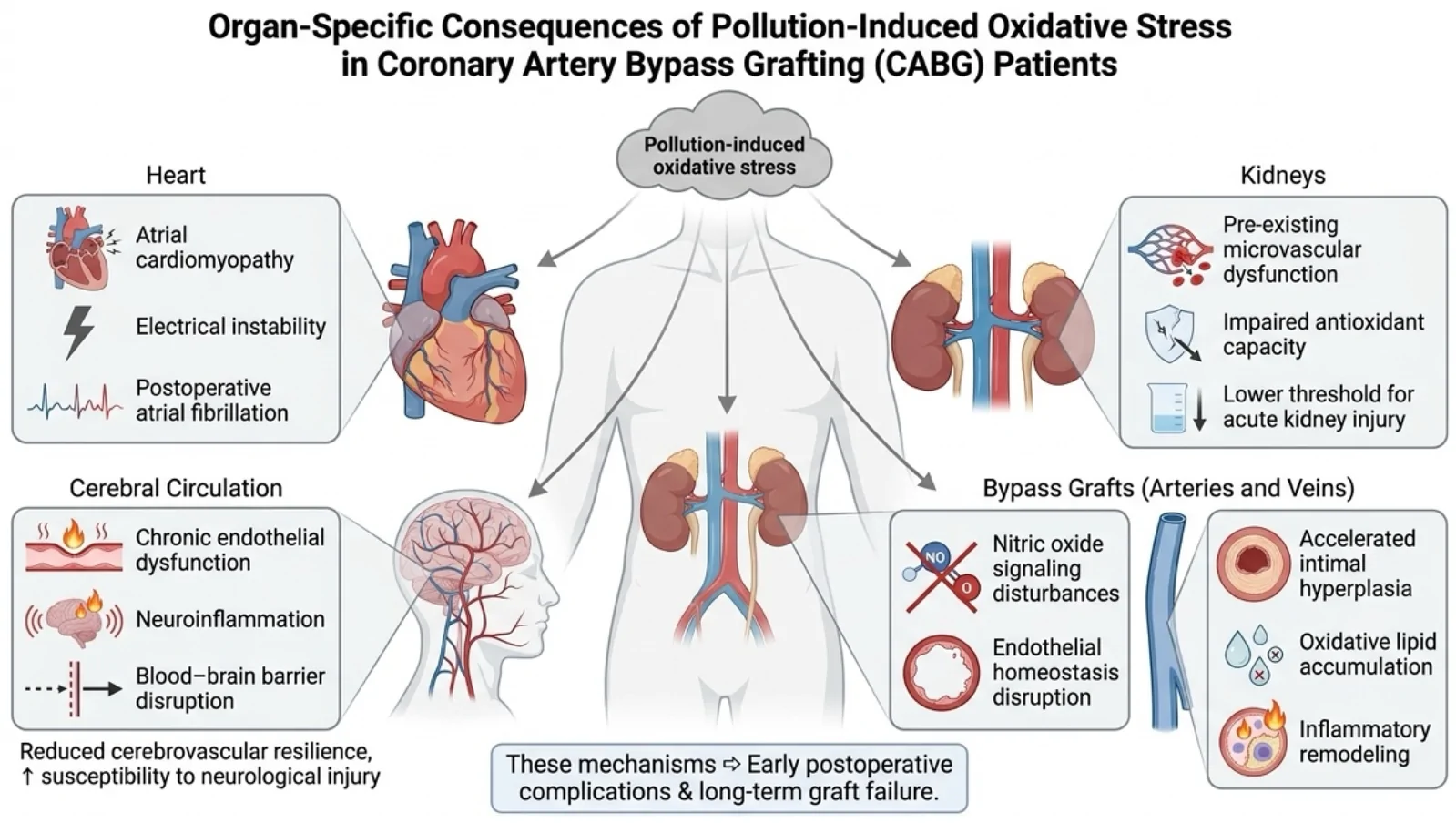
Organ-Specific Manifestations of Environmental Oxidative Priming After CABG. Created with www.figurelabs.ai (by Urbanowicz T, 2026). Arrow explanations: Solid arrows (→) represent mechanistic progression from pollution-induced oxidative stress to organ-specific pathological alterations. Diverging arrows indicate dissemination of systemic oxidative injury to the heart, cerebral circulation, kidneys, and bypass grafts. Converging arrows illustrate the cumulative contribution of these mechanisms to postoperative complications and long-term graft dysfunction. Downward arrows (↓) denote impaired protective biological functions, including antioxidant defenses and nitric oxide bioavailability. The dotted arrow (→) indicates blood–brain barrier disruption.

Not all consequences of pollution-induced oxidative stress manifest in the same manner. The biological effects of environmental exposure are ultimately expressed through specific target organs and tissues that determine postoperative recovery and long-term prognosis. The organ-specific manifestations of environmental oxidative priming, with particular emphasis on myocardial, cerebrovascular, renal, and graft-related consequences relevant to CABG, are presented in Figure 4.

The biological consequences of environmental oxidative priming are unlikely to manifest as a single complication. Rather, they may influence a spectrum of postoperative and long-term outcomes through distinct but interconnected mechanisms. Some effects become apparent within days of surgery, whereas others emerge only years after successful revascularization. Conceptualizing these outcomes according to their temporal relationship with CABG may facilitate understanding of how environmental exposure influences the entire postoperative trajectory. This framework is presented in Table 6.

These observations suggest that environmental oxidative priming should not be viewed as a uniform exposure phenomenon but rather as a biological modifier whose clinical consequences depend on the interaction between pollutant burden and the host’s intrinsic adaptive capacity. Future perioperative risk assessment may therefore benefit from integrating environmental exposure with clinical phenotypes characterized by impaired redox resilience, thereby moving from population-based estimates toward biologically informed precision risk stratification.

## 8. Postoperative Atrial Fibrillation: Is Air Pollution a Missing Driver of Atrial Vulnerability?

The preceding sections describe the molecular pathways through which chronic pollutant exposure modifies cardiovascular biology. The next question is whether these changes remain transient responses or evolve into a stable biological phenotype that influences recovery after CABG.

Postoperative atrial fibrillation (POAF) remains the most common complication following coronary artery bypass grafting, affecting approximately one-third of patients undergoing isolated CABG and substantially more patients undergoing combined procedures. Despite decades of investigation, the fundamental question remains unresolved: why do some patients develop atrial fibrillation after surgery while others exposed to apparently similar operative conditions maintain stable sinus rhythm? Traditional explanations focusing exclusively on surgical trauma, inflammation, electrolyte disturbances, and autonomic imbalance only partially account for the observed variability. Increasingly, evidence suggests that the answer may reside within the biological state of the atrium long before surgery occurs.

The conventional model of POAF assumes that cardiac surgery generates a sufficiently intense inflammatory and oxidative insult to provoke atrial arrhythmias. While this model is undoubtedly correct, it remains incomplete. Virtually all patients undergoing CABG experience ischemia–reperfusion injury, oxidative stress, and systemic inflammation, yet only a subset develop clinically significant arrhythmias. This discrepancy suggests that postoperative triggers alone are insufficient and that a susceptible atrial substrate must already be present before surgery. In contemporary electrophysiology, this substrate is increasingly described as atrial cardiomyopathy—a progressive process characterized by structural remodeling, mitochondrial dysfunction, fibrosis, inflammation, endothelial injury, and impaired cellular energetics.

Air pollution provides a biologically plausible mechanism through which such a substrate may develop. Long-term exposure to PM2.5 and traffic-related pollutants has repeatedly been associated with incident atrial fibrillation in population-based cohorts, even after adjustment for conventional cardiovascular risk factors. Large prospective population-based studies have consistently demonstrated a dose-dependent association between long-term PM2.5 exposure and incident atrial fibrillation, with most analyses reporting an approximately 10–20% increase in AF risk for every 10 μg/m^3^ increase in annual PM2.5 concentration [198,199,200]. Although the magnitude of this association varies according to exposure assessment methods, geographic region, and baseline cardiovascular risk, the overall consistency of these findings supports chronic particulate matter exposure as a clinically relevant contributor to atrial electrical remodeling.

Importantly, these associations are observed at exposure levels far below those required to produce overt cardiovascular symptoms, suggesting that pollution acts through gradual modification of atrial biology rather than through acute toxicity. The atrium, therefore, should not be viewed merely as a passive recipient of postoperative inflammation but as a tissue chronically shaped by environmental influences.

Atrial tissue appears particularly vulnerable to oxidative injury. Compared with ventricular myocardium, the atria possess thinner walls, less structural reserve, and greater sensitivity to disturbances in calcium handling and mitochondrial function. Chronic exposure to particulate matter promotes mitochondrial ROS generation, activation of redox-sensitive inflammatory pathways, and oxidative modification of intracellular proteins. Over time, these processes may alter ion channel expression, gap junction integrity, and excitation–contraction coupling. The consequence is progressive electrical instability that remains clinically silent until challenged by a sufficiently powerful trigger.

Mitochondrial dysfunction may represent the most important mechanistic link between environmental exposure and atrial arrhythmogenesis. Increasing evidence suggests that mitochondrial abnormalities are central to both pollution-induced cardiovascular injury and postoperative atrial fibrillation. Chronic particulate exposure impairs oxidative phosphorylation, disrupts mitochondrial dynamics, and increases mitochondrial ROS production. Similar abnormalities occur during ischemia–reperfusion injury associated with CABG. Consequently, patients with long-standing pollution exposure may enter surgery with pre-existing deficits in mitochondrial reserve. When confronted with the acute energetic demands of cardiac surgery, these deficits may become clinically apparent through electrical instability and arrhythmia.

An equally important but often overlooked mechanism involves nitric oxide signaling. A healthy atrium depends on intact endothelial function and adequate nitric oxide bioavailability to maintain vascular homeostasis, limit inflammation, and preserve electrophysiological stability. Air pollution disrupts this system through oxidative degradation of nitric oxide and uncoupling of endothelial nitric oxide synthase. Similar disturbances occur during cardiopulmonary bypass and reperfusion injury. The cumulative consequence may be profound microvascular dysfunction within atrial tissue, creating conditions favorable for oxidative injury, fibrosis, and arrhythmogenesis.

The concept of environmental oxidative priming may be especially relevant in patients with impaired redox homeostasis. Diabetes mellitus, obesity, chronic kidney disease, COPD, and advanced age all promote oxidative stress and atrial remodeling independently. Air pollution acts upon these same pathways, potentially accelerating progression toward a vulnerable atrial phenotype. This observation may explain why traditional clinical risk factors for POAF conditions are also strongly associated with heightened sensitivity to environmental oxidative stress. Rather than representing independent predictors, they may be manifestations of a shared biological architecture.

Perhaps the most compelling evidence supporting this perspective comes from studies of oxidative stress biomarkers. Increased atrial NADPH oxidase activity, elevated markers of lipid peroxidation, and a higher systemic oxidative stress burden have all been associated with the development of POAF after cardiac surgery. Importantly, many of these same biomarkers are elevated following chronic exposure to particulate matter and nitrogen oxides. Thus, the molecular signature of pollution exposure closely resembles that of patients who subsequently develop postoperative atrial fibrillation.

Although incorporation of environmental exposure into routine perioperative assessment remains premature, the concept of environmental oxidative priming provides a useful framework for future risk stratification strategies. Rather than viewing air pollution solely as a population-level cardiovascular risk factor, environmental exposure may be considered a determinant of individual biological resilience that influences susceptibility to surgical stress. Integration of environmental exposure metrics with biomarkers of oxidative stress, inflammation, endothelial dysfunction, and mitochondrial injury may ultimately permit identification of patients exhibiting a vulnerable preoperative oxidative phenotype. 

The proposed life-course model is conceptually based on established principles of life-course epidemiology, which recognize that cumulative environmental exposures acting throughout life progressively modify disease susceptibility and clinical outcomes. Rather than representing a statistical life-course analysis, the present framework is intended to illustrate how prolonged oxidative stress may shape biological resilience before CABG.

**Figure 5:**
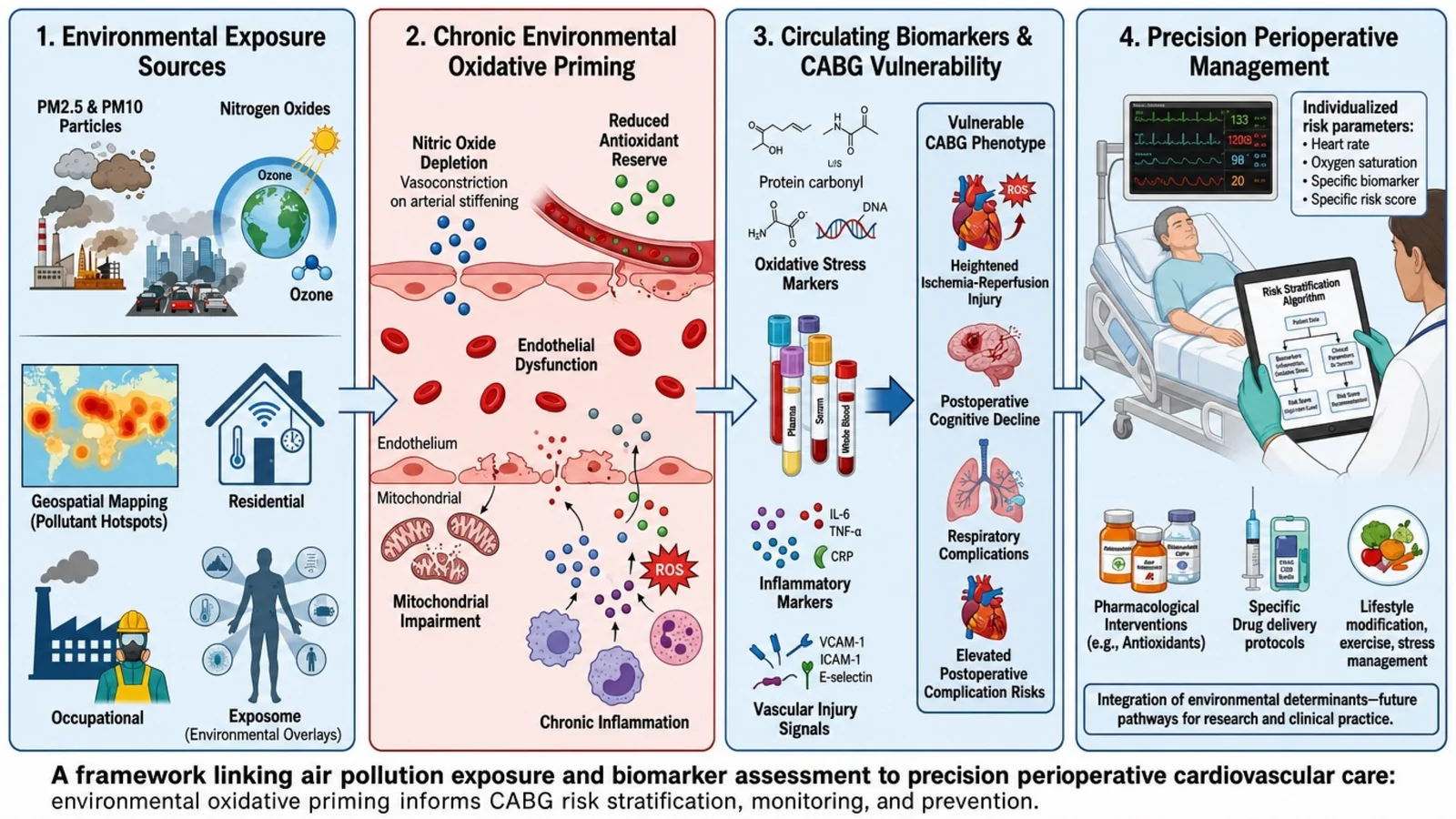
Clinical Translation of Environmental Oxidative Priming: A Framework for Environmental Risk Stratification in CABG. Created with www.figurelabs.ai (by Urbanowicz T, 2026). Arrow explanations: Solid arrows (→) represent causal and mechanistic progression across the four stages of the proposed framework, from environmental exposure to precision perioperative management. Vertical arrows indicate downstream biological processes within each stage. Converging arrows illustrate the integration of multiple pathogenic mechanisms into an individualized risk profile. The overall directional flow emphasizes translating environmental oxidative stress into biomarker-informed precision cardiovascular care. Abbreviations: CABG, coronary artery bypass grafting; CKD, chronic kidney disease; hsCRP, high-sensitivity C-reactive protein; IL-6, interleukin-6; MPO, myeloperoxidase; Nrf2, nuclear factor erythroid 2-related factor 2; NO, nitric oxide; NOx, nitrogen oxides; OxLDL, oxidized low-density lipoprotein; PM, particulate matter.

Such an approach could facilitate more personalized perioperative management, improve prediction of postoperative complications, and support the development of precision-based cardiovascular surgery. A conceptual framework illustrating the potential clinical translation of environmental oxidative priming is presented in Figure 5.

Environmental oxidative priming is best viewed as a modifier of biological resilience rather than an isolated cardiovascular risk factor. The interaction between cumulative environmental exposure and the host’s adaptive capacity may therefore help explain why patients with apparently similar clinical characteristics experience markedly different responses to an equivalent surgical insult. This concept provides the rationale for integrating environmental exposure into future biologically informed perioperative risk stratification.

## 9. Stroke and Neurological Injury: Does Air Pollution Lower the Threshold for Cerebral Injury After CABG?

The mechanistic pathways discussed in the preceding sections suggest that environmental oxidative priming is not simply another cardiovascular risk factor but a determinant of biological resilience. The remaining challenge is to translate this concept into clinically meaningful tools capable of identifying patients at increased perioperative vulnerability.

Traditionally, postoperative stroke has been viewed primarily through a procedural lens. Aortic atherosclerosis, embolization during cannulation and cross-clamping, cardiopulmonary bypass, cerebral hypoperfusion, and postoperative atrial fibrillation are recognized contributors to neurological injury. This paradigm has generated important advances in surgical technique, including anaortic approaches, epiaortic ultrasound, cerebral monitoring, and improvements in perfusion strategies. Yet a fundamental observation remains difficult to explain: embolic phenomena are common during cardiac surgery, whereas clinically apparent stroke is comparatively uncommon. This discrepancy suggests that embolic burden alone cannot fully account for neurological outcomes. Increasingly, evidence indicates that the biological condition of the cerebral circulation before surgery may be equally important. 

The blood–brain barrier represents another potential point of convergence between environmental exposure and cardiac surgery. Increasing evidence suggests that particulate matter exposure disrupts blood–brain barrier integrity through oxidative and inflammatory mechanisms. Experimental models have demonstrated increased permeability, microglial activation, oxidative DNA damage, and neurovascular dysfunction following prolonged exposure to pollutants [201,202]. These findings challenge the traditional assumption that the central nervous system is largely protected from environmental influences.

Cardiac surgery induces many of the same biological responses. The same mechanisms influence blood–brain barrier function. Consequently, patients entering surgery with pollution-induced disruption of neurovascular homeostasis may be particularly susceptible to secondary injury during the perioperative period.

Mitochondrial dysfunction provides an additional mechanistic link. Neurons possess exceptionally high metabolic demands and rely heavily on efficient mitochondrial function to maintain ionic gradients and cellular viability. Air pollution impairs mitochondrial respiration, increases mitochondrial ROS production, and promotes neuroinflammatory signaling. Similar disturbances occur during cerebral ischemia and reperfusion. Thus, chronic environmental exposure may create a state of energetic vulnerability in which neural tissue becomes less capable of tolerating ischemic stress.

An equally important but frequently overlooked issue concerns silent neurological injury. Modern neuroimaging studies have revealed that clinically overt stroke represents only a fraction of perioperative cerebral injury. Silent cerebral infarctions, microvascular ischemic lesions, and postoperative cognitive dysfunction occur substantially more frequently and may contribute to long-term cognitive decline. These outcomes are particularly relevant because they align closely with mechanisms implicated in pollution-related neurovascular disease.

Indeed, the relationship between air pollution and cognitive decline has become one of the most rapidly expanding areas of environmental health research. Chronic exposure to PM2.5 has been associated with accelerated cognitive aging, white matter injury, neuroinflammation, and increased risk of dementia. Although these observations originate primarily from non-surgical populations, they raise an important possibility: the neurological consequences of CABG may extend beyond overt stroke and may be influenced by environmental exposures that have shaped cerebral biology long before surgery.

The interaction between air pollution and neurological injury is likely to be particularly important in susceptible populations. Aging, diabetes mellitus, chronic kidney disease, frailty, and pre-existing cerebrovascular disease are all characterized by endothelial dysfunction, microvascular injury, mitochondrial impairment, and chronic inflammation. Air pollution acts upon the same biological pathways. Consequently, environmental exposure may function as a force multiplier, amplifying vulnerabilities already present within the cerebral circulation.

Large epidemiological studies and meta-analyses consistently demonstrate a dose-dependent relationship between chronic PM2.5 exposure and ischemic stroke risk [203,204]. Overall, each 10 μg/m^3^ increase in long-term PM2.5 concentration has been associated with an approximately 8–15% higher risk of ischemic stroke, with stronger associations observed among older individuals and patients with established cardiovascular disease.

Large prospective cohorts have reported approximately 6–12% higher cardiovascular mortality for each 10 μg/m^3^ increment in long-term PM2.5 exposure [205].

Observational cohort studies consistently report hazard ratios ranging from approximately 1.10 to 1.30 for major cardiovascular outcomes associated with higher long-term PM2.5 exposure, although effect estimates vary according to population characteristics, exposure assessment, and clinical endpoints [206,207].

Although quantitative evidence remains limited, observational studies consistently demonstrate higher odds of postoperative acute kidney injury among patients residing in regions with greater long-term particulate matter exposure.

Ultimately, the clinical value of environmental oxidative priming will depend not on demonstrating that pollution influences postoperative outcomes, but on determining whether incorporation of environmental biology improves prediction, procedural decision-making, or perioperative management beyond existing clinical models. If validated, this framework could shift perioperative cardiovascular medicine from exposure-based epidemiology toward biologically informed precision surgery.

## 10. Long-Term Outcomes After CABG: Persistent Oxidative Pressure and the Erosion of Surgical Benefit

**Figure 6:**
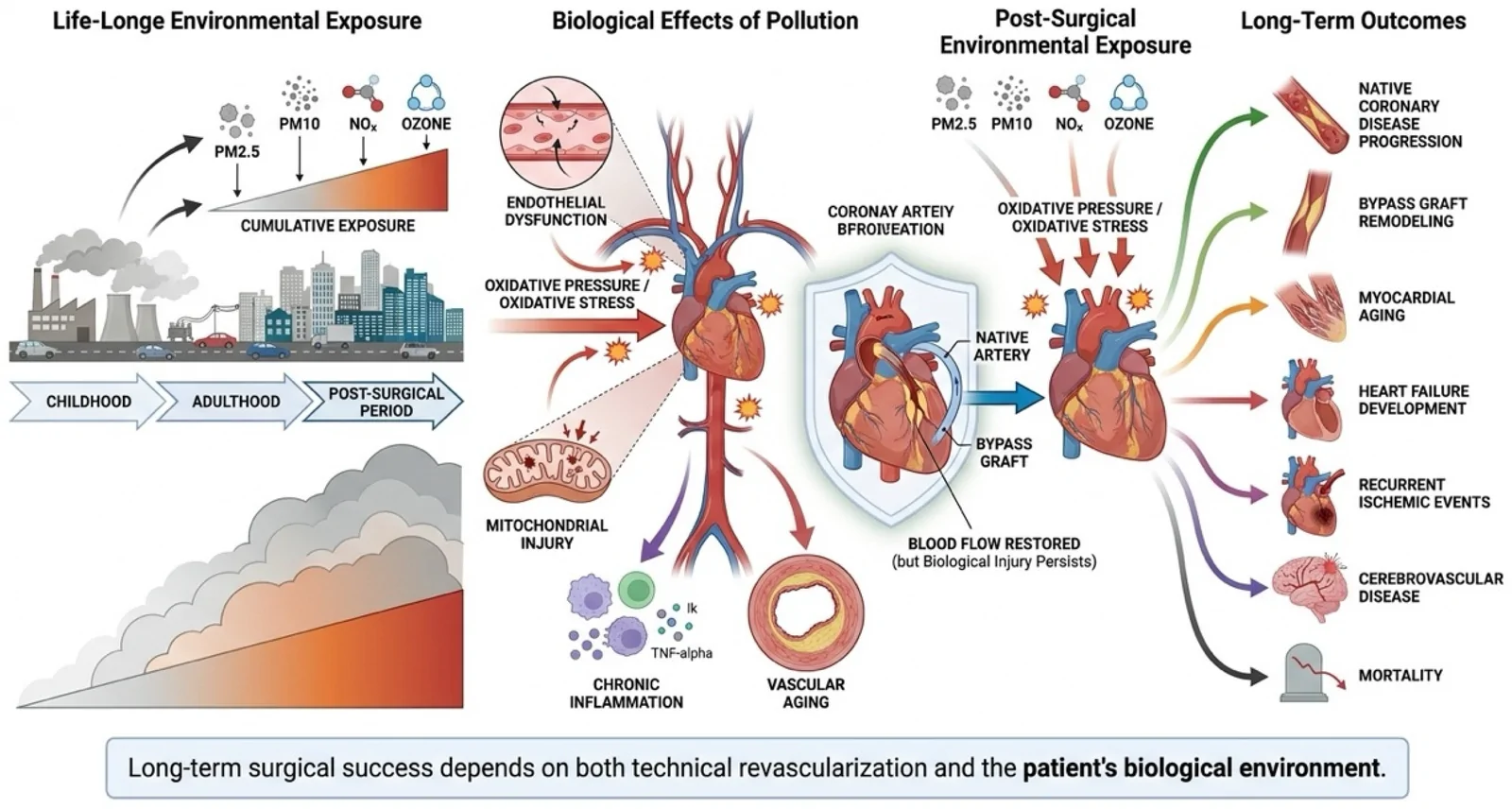
From Air Pollution Exposure to Outcome: Environmental Oxidative Pressure Across the CABG Timeline. Created by www.figurelabs.ai (by Urbanowicz T, 2026). Arrow explanations: Solid arrows (→) represent causal and temporal progression from cumulative environmental exposure to biological remodeling, perioperative oxidative stress, and long-term clinical consequences following CABG. Curved arrows illustrate cumulative lifetime exposure and persistent biological effects after surgical revascularization. Converging arrows indicate integration of multiple oxidative and inflammatory mechanisms into a shared state of environmental oxidative priming. Colored arrows represent the evolution toward specific long-term cardiovascular outcomes. Downward arrows (↓), when present, denote impaired endogenous antioxidant capacity and nitric oxide signaling. Abbreviations: CABG, coronary artery bypass grafting; MACE, major adverse cardiovascular events; ROS, reactive oxygen species.

The success of coronary artery bypass grafting is often evaluated through immediate postoperative outcomes, graft patency rates, and short-term survival [208,209]. Such metrics are undeniably important, yet they capture only part of the biological reality. CABG is not a cure for atherosclerotic disease. Rather, it is a highly effective anatomical intervention within the cardiovascular system that remains biologically active long after surgical revascularization is complete. Figure 6 illustrates the concept of persistent oxidative pressure, emphasizing how continuous environmental exposure may influence vascular aging, graft remodeling, and cardiovascular risk long after technically successful revascularization.

Among these factors, chronic air pollution deserves particular attention because it provides a sustained source of oxidative pressure throughout the postoperative period.

### 10.1. The Concept of Persistent Oxidative Pressure

Most discussions of oxidative stress in cardiac surgery focus on the perioperative period. Ischemia–reperfusion injury, cardiopulmonary bypass, and postoperative inflammation are viewed as discrete events that generate transient oxidative damage. While this perspective is appropriate for understanding early complications, it may be insufficient for explaining long-term outcomes. Air pollution represents a fundamentally different form of oxidative stress. Unlike the transient oxidative injury associated with surgery, environmental exposure persists for years or decades and continues after hospital discharge, as patients return to the same residential, occupational, and transportation environments.

### 10.2. CABG Corrects Anatomy, Not Biology

A recurring misconception in cardiovascular medicine is that successful revascularization necessarily restores cardiovascular health. In reality, CABG addresses the consequences of advanced coronary artery disease rather than the biological processes responsible for its development. The vascular abnormalities induced by chronic exposure to pollution—including endothelial dysfunction, impaired nitric oxide signaling, mitochondrial injury, chronic inflammation, and oxidative lipid modification—persist after surgery. These processes continue to influence native coronary arteries, bypass grafts, microvascular function, myocardial remodeling, and systemic vascular aging. They also influence bypass grafts, microvascular function, myocardial remodeling, and systemic vascular aging. Consequently, the patient who leaves the operating room remains exposed to many of the same pathogenic pathways that contributed to coronary disease in the first place. This may help explain why recurrent cardiovascular events continue to occur despite technically successful revascularization and aggressive secondary prevention. 

### 10.3. Air Pollution and Accelerated Vascular Aging

One of the most compelling concepts emerging from environmental cardiovascular research is that air pollution may accelerate the biological aging of the vasculature. Chronic exposure to PM2.5 and traffic-related pollutants has been linked to endothelial senescence, telomere attrition, mitochondrial dysfunction, arterial stiffening, impaired vascular repair, and progressive depletion of endothelial progenitor cell function. These observations are particularly relevant to CABG populations because many postoperative complications occurring years after surgery can be interpreted as manifestations of accelerated vascular aging. Progressive native coronary disease, graft attrition, recurrent ischemia, heart failure, cerebrovascular disease, and declining functional capacity all reflect deterioration of vascular and myocardial resilience over time. 

### 10.4. Oxidative Stress and the Progression of Residual Cardiovascular Disease

Even after complete surgical revascularization, substantial cardiovascular disease remains. Atherosclerosis continues to affect native coronary arteries, cerebral vessels, peripheral arteries, the aorta, and microvascular networks throughout the body. The progression of this residual disease is heavily influenced by oxidative stress. Reactive oxygen species promote oxidation of lipoproteins, endothelial activation, inflammatory cell recruitment, vascular smooth muscle proliferation, extracellular matrix remodeling, and plaque instability. These processes are not confined to the coronary circulation and therefore cannot be fully addressed through revascularization alone. Because these mechanisms extend far beyond the surgically treated coronary segments, revascularization alone cannot halt their progression. Continued exposure to air pollution may therefore sustain residual cardiovascular disease and contribute to recurrent myocardial infarction, stroke, peripheral arterial disease, and repeat revascularization.

### 10.5. Heart Failure: The Forgotten Long-Term Outcome

Although long-term outcome studies traditionally emphasize graft patency and recurrent ischemia, heart failure may represent an equally important determinant of prognosis. Pollution-induced mitochondrial injury may influence myocardial recovery after revascularization by limiting energetic reserve, promoting fibrosis, and impairing adaptive responses to ischemia. These effects may be particularly relevant in patients with reduced ejection fraction, diabetes, advanced age, or extensive myocardial scar burden. In such patients, long-term prognosis may depend as much on preservation of mitochondrial function as on the durability of grafts themselves.

### 10.6. Major Adverse Cardiovascular Events as a Manifestation of Biological Resilience

Traditionally, major adverse cardiovascular events (MACE) are treated as discrete clinical outcomes. Myocardial infarction, stroke, heart failure hospitalization, repeat revascularization, and cardiovascular death may all be viewed as manifestations of declining biological resilience within a chronically stressed cardiovascular system. This perspective is particularly relevant to environmental exposures. Air pollution does not target a single organ, vessel, or disease pathway. It influences systemic mechanisms that govern adaptation, repair, and recovery throughout the cardiovascular system. Consequently, environmental exposure may influence several cardiovascular endpoints simultaneously. Their shared biological substrate is likely the progressive decline in adaptive vascular and myocardial resilience rather than a single disease-specific mechanism.

### 10.7. Environmental Memory and Long-Term Prognosis

Environmental exposures leave durable molecular signatures involving epigenetic regulation, mitochondrial function, inflammatory programming, and endothelial phenotype. These changes may persist long after individual pollution peaks have subsided. Patients therefore carry not only a history of environmental exposure but also its persistent molecular imprint. Future studies should therefore move beyond contemporary pollution measurements and instead examine cumulative lifetime exposure as a determinant of postoperative cardiovascular aging. Such an approach aligns with the emerging concept of the cardiovascular exposome and may provide a more comprehensive understanding of long-term prognosis after surgical revascularization.

### 10.8. Reframing Long-Term Success After CABG

If environmental oxidative priming influences perioperative outcomes, persistent environmental oxidative pressure may determine how long those benefits endure. Long-term success after CABG may therefore depend not only on the operation itself but also on the environment into which the patient returns. In this sense, the story of CABG does not end at hospital discharge but continues through the dynamic interaction between vascular repair and ongoing environmental injury, in which chronic air pollution may play a greater role than currently appreciated.

Because environmental oxidative priming involves multiple interconnected biological processes, candidate biomarkers can be organized into four principal domains: oxidative stress, endothelial dysfunction, inflammation, and mitochondrial injury. Such pathway-oriented classification facilitates biological interpretation and may assist future translational studies investigating perioperative susceptibility following CABG.

Environmental oxidative priming is unlikely to be adequately characterized by a single circulating biomarker. Instead, it reflects coordinated disturbances across multiple biological domains, including oxidative stress, endothelial dysfunction, inflammation, mitochondrial injury, and vascular remodeling. Accordingly, the candidate biomarkers summarized in Table 7 are organized by their predominant mechanistic pathways rather than by their individual diagnostic performance. These biomarkers should be regarded as candidates for future hypothesis-driven prospective studies rather than as validated clinical tools, and their translational value will require confirmation in appropriately designed cohorts that incorporate rigorous statistical correction for multiple testing.

## 11. Therapeutic Modulation of Pollution-Induced Oxidative Stress: Opportunities and Limitations

If air pollution contributes to adverse outcomes after coronary artery bypass grafting via oxidative mechanisms, a natural question arises: can these effects be therapeutically modified? Although conceptually appealing, the answer is considerably more complex than anticipated from decades of antioxidant research. Decades of experimental research have established oxidative stress as a central mediator of vascular injury, yet clinical trials of antioxidant therapies have generally yielded inconsistent and often disappointing results. Resolving this apparent paradox remains one of the major challenges in translational cardiovascular medicine.

Part of the explanation lies in the evolving understanding of redox biology itself. Reactive oxygen species are no longer viewed exclusively as harmful by-products of cellular metabolism. Under physiological conditions, they function as essential signaling molecules involved in vascular homeostasis, cellular adaptation, angiogenesis, immune responses, and mitochondrial communication. Consequently, indiscriminate suppression of reactive oxygen species is unlikely to represent an effective therapeutic strategy. The clinical objective is not the elimination of oxidative signaling but the preservation of redox balance.

This distinction is particularly relevant when considering pollution-induced oxidative stress. Environmental exposure does not generate a single oxidative pathway that can be neutralized pharmacologically. Instead, chronic pollutant exposure induces coordinated biological reprogramming that progressively alters vascular adaptation, mitochondrial function, inflammatory signaling, and tissue repair. The resulting phenotype reflects cumulative biological adaptation rather than an isolated biochemical abnormality.

### 11.1. The Limitations of Conventional Antioxidant Therapy

The disappointing performance of conventional antioxidant supplementation provides an instructive example. Vitamins C and E, β-carotene, and other antioxidant compounds demonstrated considerable promise in experimental models but failed to consistently improve cardiovascular outcomes in large clinical trials [219]. Several explanations have been proposed, including inadequate dosing, inappropriate patient selection, and intervention at advanced stages of disease. A more fundamental limitation may be the assumption that oxidative stress represents a single therapeutic target rather than a heterogeneous biological state.

Environmental oxidative injury evolves gradually and becomes embedded within vascular and myocardial biology. Consequently, interventions directed at a single molecular pathway are unlikely to reverse decades of cumulative biological remodeling. Rather than arguing against the importance of oxidative stress, the limited success of conventional antioxidant supplementation highlights the complexity of redox biology and the need for more targeted therapeutic strategies.

### 11.2. Endothelial Protection as a Therapeutic Target

Among the multiple pathways affected by air pollution, endothelial dysfunction may represent the most clinically actionable. Because endothelial dysfunction is a shared consequence of chronic environmental exposure and CABG, preserving endothelial integrity may confer broad therapeutic benefits. Consequently, therapies that preserve endothelial function may provide broader biological benefits than interventions directed solely at reactive oxygen species.

Several established cardiovascular therapies possess important endothelial protective properties. Statins improve nitric oxide bioavailability, reduce vascular inflammation, and attenuate oxidative stress, independent of lipid-lowering [220]. Renin–angiotensin system inhibitors similarly improve endothelial function by modulating oxidative pathways and reducing vascular inflammation [221,222]. Increasing evidence also suggests that sodium–glucose cotransporter-2 inhibitors exert favorable effects on endothelial biology and mitochondrial function [223,224]. These therapies illustrate an important therapeutic principle: modulation of upstream mechanisms governing vascular homeostasis appears more effective than indiscriminate scavenging of reactive oxygen species.

### 11.3. Mitochondrial Preservation and Energetic Resilience

Mitochondria represent another attractive therapeutic target because they integrate many of the biological consequences of both chronic pollutant exposure and surgical ischemia–reperfusion injury. Chronic exposure to particulate matter impairs mitochondrial respiration, increases mitochondrial ROS production, alters mitochondrial dynamics, and promotes inflammatory signaling. Similar disturbances occur during ischemia–reperfusion injury and cardiopulmonary bypass.

This convergence has generated interest in therapies that preserve mitochondrial function rather than directly scavenging reactive oxygen species. Mitochondrial-targeted antioxidants, coenzyme Q10, modulators of mitochondrial biogenesis, and interventions designed to improve energetic efficiency have demonstrated encouraging results in experimental studies. However, translation into clinical practice remains limited.

The principal advantage of these approaches lies in preserving mitochondrial function rather than suppressing oxidative signaling indiscriminately. Given the central role of mitochondrial integrity during ischemia–reperfusion injury, such strategies may ultimately prove more effective than conventional antioxidant supplementation.

### 11.4. Nrf2 and Endogenous Antioxidant Defense

Another important lesson emerging from contemporary redox biology is that resistance to oxidative injury depends not only on the magnitude of oxidative stress but also on the integrity of endogenous defense systems. The transcription factor Nrf2 regulates numerous antioxidant and cytoprotective pathways, including glutathione metabolism, heme oxygenase activity, and cellular detoxification mechanisms [225].

Experimental studies suggest that chronic exposure to air pollution may impair these adaptive responses, thereby reducing tissues’ capacity to tolerate subsequent oxidative challenges. This observation has shifted attention toward interventions that enhance endogenous antioxidant defenses rather than toward providing exogenous antioxidant molecules.

From a conceptual standpoint, this approach is attractive because it focuses on resilience rather than suppression. The objective is not to eliminate oxidative stress but to improve the organism’s ability to respond appropriately to oxidative challenges. Such a strategy may be particularly relevant in cardiac surgery, where complete avoidance of oxidative injury is impossible.

### 11.5. Exposure Reduction: The Most Direct Intervention

Despite increasing interest in pharmacological interventions, reducing pollutant exposure remains the most direct strategy for limiting environmentally induced oxidative injury. Evidence from environmental health studies demonstrates that improvements in air quality are associated with rapid reductions in cardiovascular morbidity and mortality [226,227]. Similarly, reductions in individual exposure through air filtration systems, avoidance of highly polluted environments, and improvements in indoor air quality have been associated with measurable improvements in endothelial function and inflammatory biomarkers.

Although such strategies remain largely unexplored in CABG populations, they address the fundamental source of environmental oxidative injury rather than its biological consequences. In this regard, exposure reduction may represent the most direct form of antioxidant therapy currently available.

### 11.6. Toward Precision Redox Medicine

Therapeutic strategies should account for substantial interindividual differences in environmental exposure and biological resilience. Future strategies may therefore require a more individualized approach integrating environmental exposure assessment, oxidative stress biomarkers, mitochondrial profiling, and clinical phenotype. Such a framework would allow identification of patients most likely to benefit from interventions targeting pollution-induced oxidative injury.

This concept, which may be described as precision redox medicine, represents a departure from traditional antioxidant paradigms. Rather than attempting to suppress oxidative stress universally, it seeks to identify specific biological vulnerabilities and intervene selectively. For patients undergoing CABG, such an approach may ultimately prove more effective than generalized antioxidant supplementation.

Although the therapeutic implications of environmental oxidative priming remain hypothetical, converging evidence from environmental medicine, vascular biology, and cardiac surgery supports the concept that oxidative injury represents a potentially modifiable determinant of postoperative resilience rather than merely a marker of disease severity.

**Figure 7:**
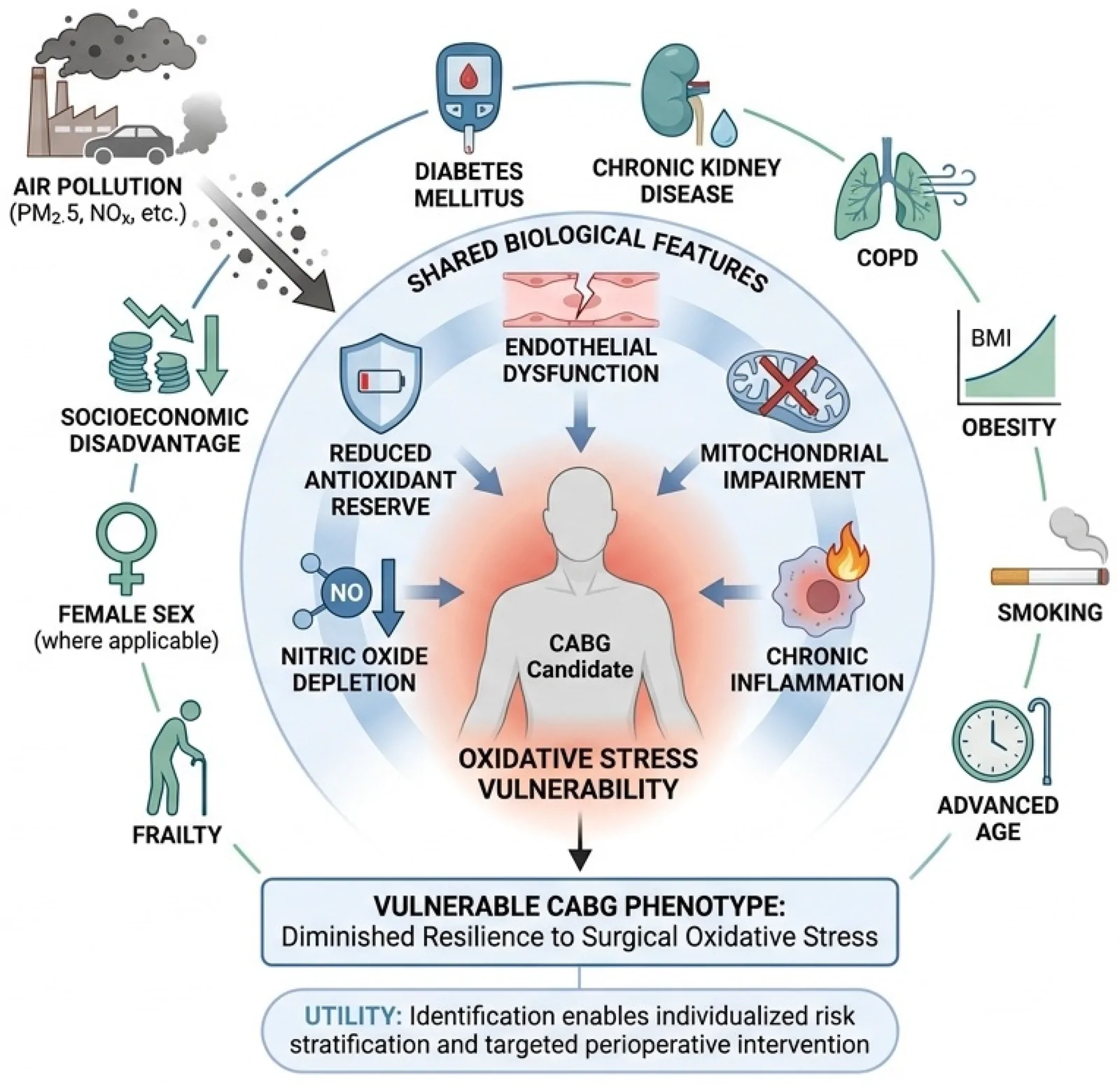
The Vulnerable CABG Phenotype: Clinical Modifiers of Environmental Oxidative Priming. Created by www.figurelabs.ai (by Urbanowicz T, 2026). Arrow explanations: Solid arrows (→) represent mechanistic progression from environmental and patient-related determinants to shared biological pathways and the vulnerable CABG phenotype. Circular arrows indicate the cumulative influence of multiple interacting risk factors. Converging arrows illustrate the integration of endothelial dysfunction, mitochondrial injury, oxidative stress, chronic inflammation, and impaired antioxidant defenses into a common biological phenotype. Downward arrows (↓) indicate depletion of endogenous protective mechanisms, including nitric oxide signaling and antioxidant reserve, ultimately contributing. Abbreviations: COPD, chronic obstructive pulmonary disease; CKD, chronic kidney disease; NO, nitric oxide.

The therapeutic implications of environmental oxidative priming depend on the ability to identify patients most biologically susceptible to its effects. Accumulating evidence suggests that pollution-induced oxidative stress does not affect all CABG patients equally but preferentially amplifies pre-existing vulnerabilities related to metabolic, pulmonary, renal, and age-associated disorders. Figure 7 summarizes the proposed vulnerable CABG phenotype by integrating environmental exposures with biological susceptibilities and highlighting potential targets for individualized perioperative interventions.

The priority for future research is therefore not simply the development of new antioxidant compounds but the identification of susceptible patients, biologically relevant therapeutic targets, and optimal windows for intervention.

## 12. From Exposure to Biology: Biomarkers, the Exposome, and the Environmental Risk Stratification in CABG

The central hypothesis of this review is that chronic air pollution exposure induces environmental oxidative priming, thereby modifying the host response to CABG and contributing to variability in early and long-term outcomes. Despite the biological plausibility of this framework, a fundamental challenge remains. Although pollutant exposure, oxidative injury, and surgical outcomes can each be quantified, the biological processes linking them remain difficult to capture in clinical practice.

This challenge reflects an important translational gap in contemporary cardiovascular medicine. Most environmental studies focus on exposure assessment, whereas most surgical investigations focus on clinical outcomes. Between these fields lies a poorly characterized intermediate phenotype that reflects vascular function, mitochondrial reserve, inflammatory responsiveness, and endogenous antioxidant capacity. It is within the intermediate phenotype that environmental exposure is translated into clinically relevant cardiovascular risk. The future of this field will therefore depend not only on identifying new pollutants or new biomarkers, but on understanding how exposure becomes biology.

### 12.1. The Limitations of Exposure-Based Risk Assessment

Current environmental studies largely rely on ambient pollutant concentrations derived from monitoring stations, satellite data, geospatial models, or residential proximity to major roads. These approaches have been instrumental in establishing associations between pollution and cardiovascular disease. However, they provide only indirect information regarding biological susceptibility.

Two individuals living within the estimated exposure may nevertheless exhibit markedly different physiological responses. Age, sex, genetic background, comorbidities, co-exposures, pulmonary function, and endogenous antioxidant capacity all modify the biological consequences of pollutant exposure. Consequently, pollutant concentration alone cannot fully explain individual risk.

This distinction is particularly important in CABG populations. The clinically relevant variable may not be exposure alone, but the extent to which it has altered vascular and cellular function before surgery. Future risk models must therefore move beyond environmental measurements and incorporate markers of biological response.

### 12.2. Why Oxidative Stress Biomarkers Have Been Disappointing

At first glance, oxidative stress biomarkers appear ideally suited to this task. Numerous studies have evaluated circulating markers of lipid, protein, and DNA oxidation, including malondialdehyde, F2-isoprostanes, oxidized low-density lipoprotein, and protein carbonyls [228,229,230,231,232,233,234,235]. Yet despite extensive investigation, few have entered routine cardiovascular practice.

The problem is not necessarily lack of biological relevance. They provide information on the current oxidative burden but offer limited insight into physiological resilience. They provide information on the current oxidative burden but offer limited insight into physiological resilience. For a patient undergoing CABG, the critical question is not simply whether oxidative stress is present, but whether sufficient reserve remains to tolerate the additional oxidative burden of surgery. This distinction mirrors the difference between measuring damage and measuring vulnerability. A truly informative biomarker would identify patients at risk before complications develop, rather than merely documenting oxidative stress after surgery.

### 12.3. Endothelial Phenotyping: Measuring the First Target of Environmental Injury

Among the systems affected by air pollution, the endothelium is particularly relevant to perioperative risk stratification. It is among the earliest targets of environmental injury and simultaneously a key determinant of postoperative recovery, graft adaptation, microvascular function, and organ perfusion.

Future studies may increasingly focus on endothelial phenotyping rather than isolated oxidative stress markers. Circulating endothelial extracellular vesicles, endothelial progenitor cell function, glycocalyx degradation products, adhesion molecules, and measures of vascular reactivity may together provide a more integrated assessment of environmentally induced vascular injury.

Such approaches are appealing because they capture the cumulative consequences of multiple oxidative and inflammatory pathways rather than focusing on a single molecular endpoint. In essence, they may offer a biological readout of environmental exposure.

### 12.4. Mitochondria as Integrators of Environmental History

Mitochondrial phenotyping offers a complementary approach to assessing cumulative environmental injury. Mitochondria respond directly to environmental pollutants, participate in redox signaling, regulate inflammatory pathways, and determine cellular energetic reserve during ischemia-reperfusion injury.

Importantly, mitochondria possess a form of biological memory. Repeated environmental insults leave durable effects on mitochondrial structure, function, and signaling. Consequently, mitochondrial phenotypes may provide insight into cumulative environmental injury in ways that transient oxidative stress markers cannot.

Potential approaches include circulating mitochondrial DNA, cellular bioenergetic profiling, assessment of respiratory reserve, and metabolomic analysis. Rather than measuring oxidative stress itself, these methods assess the biological systems that respond to it.

### 12.5. The Exposome: A Missing Dimension in Cardiac Surgery

The exposome has emerged as an important framework for integrating environmental exposures across the life course [236,237]. Analogous to the genome, the exposome encompasses the totality of environmental exposures experienced throughout life and their biological consequences.

For cardiovascular surgery, this framework extends risk assessment beyond a single preoperative time point. Traditional risk models evaluate patients at a single point in time using a limited number of clinical variables. The exposome recognizes that biological vulnerability is continuously shaped by interactions among environmental exposures, lifestyle factors, social determinants, and physiological adaptation.

Air pollution is only one component of this broader framework. Other relevant components include noise, occupational exposures, diet, physical activity, psychosocial stress, housing conditions, and socioeconomic disadvantage, each of which may influence redox biology and cardiovascular health [238]. The biological state of a patient presenting for CABG, therefore, reflects a lifetime of accumulated exposures rather than a snapshot of contemporary clinical variables. The principal challenge is to translate this complexity into clinically interpretable risk models.

### 12.6. Toward Environmental Precision Cardiovascular Surgery

The ultimate objective is to translate exposure and biomarker data into individualized risk prediction. The current surgical risk models implicitly assume that patients with similar demographic and clinical characteristics possess comparable biological resilience. Environmental research increasingly suggests otherwise. Patients with similar STS or EuroSCORE II estimates may nevertheless differ substantially in endothelial reserve, mitochondrial function, inflammatory responsiveness, and antioxidant capacity because of differences in lifelong exposure. These observations support the concept of environmental precision cardiovascular surgery. Within such a framework, risk assessment would extend beyond conventional clinical variables to incorporate measures of environmental exposure and biological susceptibility. If prospectively validated, such profiles could identify patients who warrant intensified monitoring, targeted preventive measures, or individualized perioperative management. Clinical implementation remains uncertain and will require prospective validation. Nevertheless, convergence among environmental science, redox biology, and cardiac surgery increasingly supports the inclusion of environmental factors in perioperative research.

### 12.7. Beyond Biomarkers: Measuring Biological Resilience

Future progress may depend on shifting the focus from oxidative stress alone to biological resilience. The question is not simply who has elevated oxidative biomarkers, but who possesses diminished capacity to withstand the oxidative challenge of surgery.

Air pollution provides a relevant model for this concept because cumulative exposure can progressively modify vascular, mitochondrial, inflammatory, and adaptive reserve before surgery. CABG then reveals the clinical consequences of these processes through postoperative complications, graft adaptation, and long-term outcomes.

In this sense, the future of environmental cardiovascular medicine may not lie in identifying a single biomarker or a single pollutant. It may lie in understanding how environmental exposures become biologically embedded and how that biological memory influences resilience to major physiological stress. For patients undergoing CABG, this knowledge could complement technical and clinical advances by improving understanding of interindividual susceptibility to surgical stress.

## 13. Alternative Interpretations, and a Critical Perspective

Any framework linking air pollution-induced oxidative stress to outcomes after coronary artery bypass grafting must be interpreted with caution. The hypothesis of environmental oxidative priming is biologically plausible and consistent with current knowledge of vascular and mitochondrial biology, but plausibility is not proof. Several important limitations and alternative interpretations deserve explicit consideration.

### 13.1. The Fundamental Problem of Causality

The strongest evidence connecting air pollution to cardiovascular disease comes from epidemiological studies. These studies consistently demonstrate associations between PM2.5, PM10, NO_2_, and adverse cardiovascular outcomes. However, observational associations—even highly consistent ones—do not establish causality at the individual level. Patients living in areas with high pollution often differ from those in less polluted areas in ways that extend beyond environmental exposure. Socioeconomic status, diet, healthcare access, occupational hazards, psychosocial stress, physical activity, and smoking patterns may all contribute to the observed associations.

In CABG populations, this challenge is magnified. Patients residing in highly polluted environments frequently present a greater burden of diabetes, obesity, chronic kidney disease, COPD, and other comorbidities that independently contribute to oxidative stress and adverse surgical outcomes. Disentangling environmental effects from the broader social and biological context remains difficult.

### 13.2. Exposure Is Not Biology

A second limitation is the assumption that ambient pollutant concentration accurately reflects biological exposure. Most environmental studies estimate exposure using residential location, monitoring stations, or satellite data. Yet individuals spend substantial time indoors, commute through different environments, and experience highly variable personal exposures. More importantly, identical exposures do not produce identical biological responses.

The clinically relevant variable may therefore be biological susceptibility rather than pollutant concentration itself. A patient with robust endothelial function and preserved antioxidant reserve may tolerate environmental exposure differently from a patient with diabetes, frailty, or chronic inflammation. Current exposure metrics more accurately characterize environmental conditions than individual biological exposure or susceptibility.

### 13.3. Oxidative Stress Is an Attractive but Incomplete Explanation

Oxidative stress provides a convenient unifying mechanism because it connects pollution, atherosclerosis, ischemia-reperfusion injury, inflammation, and organ dysfunction. However, there is a risk of turning oxidative stress into a catch-all explanation for complex biological phenomena. Many pathways implicated in CABG outcomes—including immune activation, coagulation, autonomic dysfunction, metabolic signaling, and tissue repair—cannot be fully reduced to reactive oxygen species generation.

Many adverse effects traditionally attributed to oxidative stress may instead reflect broader disturbances in mitochondrial function, endothelial signaling, immune regulation, and cellular adaptation. Distinguishing primary redox-mediated injury from secondary oxidative responses remains an important challenge for future research.

### 13.4. The Antioxidant Paradox

Perhaps the most revealing limitation is the longstanding discrepancy between mechanistic evidence and therapeutic success. If oxidative stress is central to cardiovascular injury, why have conventional antioxidant therapies generally failed to improve major clinical outcomes? This paradox cannot be ignored.

One interpretation is that oxidative stress is important but therapeutically inaccessible through simple scavenging strategies. Another is that oxidative stress represents a downstream consequence of more fundamental disturbances in mitochondrial function, endothelial signaling, inflammation, or metabolism. An alternative explanation is that therapeutic responses observed in experimental models may not translate directly to the biological heterogeneity encountered in clinical populations.

For the present framework, this means that identifying pollution-induced oxidative stress does not automatically identify a viable therapeutic target.

### 13.5. A Question of Scale: Molecular Signals Versus Clinical Events

Another critical issue concerns scale. Many studies demonstrate measurable changes in oxidative biomarkers following pollution exposure. Yet the relationship between molecular perturbations and major clinical outcomes such as stroke, graft failure, heart failure, or death is often indirect. Statistically detectable alterations in oxidative biomarkers do not necessarily translate into clinically meaningful differences in postoperative outcomes.

Conversely, catastrophic postoperative events frequently result from multiple converging processes rather than a single dominant mechanism. Air pollution may contribute to vulnerability without being the principal determinant of outcome in many individual patients.

### 13.6. The Danger of Environmental Determinism

An additional concern is the risk of environmental determinism—the implication that patients exposed to pollution are biologically destined to experience worse outcomes. Such a conclusion would be neither scientifically justified nor clinically helpful [239].

Environmental exposure should be viewed as one contributor within a complex network of genetic, metabolic, behavioral, and social factors. Its effects are likely probabilistic rather than deterministic. Many heavily exposed individuals maintain good cardiovascular health, while some individuals with relatively low exposure develop severe disease. The objective of this framework is to identify an additional dimension of risk, not to replace established cardiovascular biology.

### 13.7. What Would Falsify the Hypothesis?

A useful scientific framework should be testable and potentially falsifiable. The environmental oxidative priming hypothesis would be weakened if future studies demonstrated that:

1. Pollution exposure is not associated with measurable differences in endothelial, mitochondrial, or oxidative phenotypes among CABG patients.
2. Patients with high environmental exposure do not exhibit greater susceptibility to oxidative stress-related complications after adjustment for conventional risk factors.
3. Biomarkers of pollution-related oxidative injury fail to predict postoperative or long-term outcomes.
4. Interventions that reduce exposure or improve redox resilience do not influence biological or clinical endpoints in high-exposure populations.

Addressing these questions will determine whether environmental oxidative priming represents a clinically meaningful biological process or remains an attractive but incompletely supported hypothesis.

Despite substantial advances in environmental cardiovascular science, important gaps remain in our understanding of how chronic exposure to pollution influences outcomes after coronary artery bypass grafting. Most available evidence comes from epidemiological studies conducted in non-surgical populations, while direct mechanistic and clinical investigations remain scarce. Identifying these knowledge gaps is essential for guiding future research and translating environmental cardiovascular biology into perioperative practice. The major unresolved questions and potential investigative strategies are summarized in Table 8.

### 13.8. Future Directions—What Can We Change?

At present, the evidence does not justify the conclusion that air pollution is a major independent determinant of CABG outcomes. A more defensible interpretation is that air pollution may contribute to a broader state of impaired biological resilience characterized by endothelial dysfunction, mitochondrial injury, chronic inflammation, and reduced adaptive reserve.

Traditionally, perioperative risk assessment has focused on anatomical complexity, operative variables, and patient comorbidities. The environmental oxidative priming framework expands this perspective by recognizing that cumulative lifelong environmental exposures may influence endothelial function, mitochondrial integrity, inflammatory regulation, and tissue repair before surgery occurs. Consequently, environmental exposure should be viewed not as an isolated determinant of postoperative outcomes but as one component of an individual’s broader biological resilience.

The concept of environmental oxidative priming introduces a broader perspective in which surgical outcomes are viewed not solely as consequences of operative events, but as the culmination of lifelong interactions between the individual and the environment. From this standpoint, the biological response to CABG is shaped not only by age, ventricular function, diabetes, or renal impairment, but also by the cumulative environmental exposures that have influenced vascular resilience, inflammatory regulation, mitochondrial competence, and tissue repair capacity over decades.

Importantly, the relevance of this concept extends beyond air pollution itself. Environmental exposure may represent one component of a larger exposomic framework encompassing the totality of non-genetic influences acting throughout life. Such a perspective encourages a transition from conventional risk prediction toward a more integrated understanding of biological susceptibility. Rather than asking whether two patients have identical operative risk profiles, future perioperative medicine may increasingly ask whether they possess comparable capacities to withstand physiological stress.

If environmental oxidative priming contributes meaningfully to outcome variability after CABG, its implications extend across the entire continuum of care. Environmental information could become relevant not as an isolated risk factor, but as part of a broader effort to characterize individual biological resilience, refine procedural selection, guide perioperative management, and identify patients most likely to benefit from targeted preventive strategies. The potential clinical and research implications of this emerging paradigm are summarized in Table 9.

The proposed model should therefore be considered a biologically plausible mechanistic framework rather than definitive clinical evidence. Future prospective cohorts integrating environmental exposure assessment with oxidative biomarkers and surgical outcomes will be required to validate the concept of environmental oxidative priming.

## 14. Limitations

Several limitations should be acknowledged when interpreting the concepts presented in this review. First, the proposed framework of environmental oxidative priming remains primarily hypothesis-generating. Although substantial evidence supports independent associations between air pollution, oxidative stress, endothelial dysfunction, mitochondrial injury, and adverse cardiovascular outcomes, direct evidence linking long-term environmental exposure to specific outcomes after coronary artery bypass grafting remains limited. Much of the mechanistic rationale presented herein is therefore derived from the integration of environmental health, vascular biology, and cardiac surgical literature rather than from studies specifically designed to evaluate CABG populations. Second, air pollution exposure is inherently difficult to quantify at the individual level. Most epidemiological studies rely on regional monitoring stations, geospatial models, satellite-derived estimates, or residential location data, which may not accurately reflect personal exposure. Important determinants such as occupational exposures, commuting patterns, indoor air quality, socioeconomic circumstances, smoking history, and residential mobility may substantially modify cumulative pollutant burden and introduce exposure misclassification. Third, the biological consequences of environmental exposure demonstrate considerable interindividual variability. Genetic background, age, sex, nutritional status, comorbid disease, pulmonary function, antioxidant capacity, and medication use all influence susceptibility to oxidative injury. Consequently, similar environmental exposures may produce markedly different biological responses among individual patients. Current approaches to exposure assessment do not adequately capture this heterogeneity. Fourth, oxidative stress itself remains challenging to measure in clinical practice. Although numerous biomarkers have been proposed, including malondialdehyde, F2-isoprostanes, oxidized low-density lipoprotein, and inflammatory mediators, no single biomarker reliably reflects the complex and dynamic nature of redox biology. Most available markers quantify oxidative injury after it has occurred rather than the underlying susceptibility or resilience that may determine postoperative outcomes. Fifth, several of the associations discussed throughout this review may be influenced by residual confounding. Air pollution is closely linked to socioeconomic status, urbanization, healthcare access, lifestyle behaviors, occupational exposures, and other environmental factors that independently affect cardiovascular outcomes. Although many epidemiological studies adjust for these variables, complete elimination of confounding remains difficult and causal inference should therefore be interpreted with caution. Sixth, the mechanistic pathways highlighted in this review—including endothelial dysfunction, mitochondrial injury, nitric oxide depletion, NADPH oxidase activation, Nrf2 dysregulation, and chronic inflammation—are not unique to air pollution. These processes are also influenced by diabetes mellitus, chronic kidney disease, obesity, smoking, aging, and other cardiovascular risk factors frequently encountered in CABG populations. Distinguishing pollution-specific effects from broader determinants of oxidative stress remains an important challenge for future investigations. Finally, the clinical implications discussed herein should not be interpreted as evidence supporting immediate changes in surgical decision-making or perioperative management. At present, environmental exposure assessment is not incorporated into established cardiac surgical risk models, and no prospective studies have demonstrated that modifying pollution exposure alters CABG outcomes. Future research integrating environmental exposure metrics, biomarker profiling, mechanistic investigation, and long-term clinical follow-up will be necessary to determine whether environmental oxidative priming represents a clinically actionable determinant of postoperative risk.

Despite these limitations, the convergence of evidence from environmental cardiovascular medicine, redox biology, and cardiac surgery provides a biologically plausible foundation for further investigation. The concept of environmental oxidative priming should therefore be viewed not as a definitive explanation for variability in CABG outcomes, but as a framework intended to stimulate future mechanistic, translational, and clinical research.

## 15. Conclusions: Reframing CABG Through the Lens of Environmental Oxidative Priming

Coronary artery bypass grafting is traditionally viewed as a technical solution to an anatomical problem. Surgical success is commonly evaluated through procedural outcomes, graft patency, freedom from ischemia, and survival. While this perspective has driven remarkable advances in cardiovascular surgery, it may overlook an important biological reality: patients enter surgery with distinct biological characteristics shaped by aging, genetics, metabolic disease, lifestyle, and lifelong environmental exposures. Rather, they arrive carrying the cumulative consequences of aging, genetics, metabolic disease, lifestyle, and lifelong environmental exposures. Among these influences, air pollution has emerged as one of the most pervasive and biologically active determinants of cardiovascular health.

The central premise of this review is that chronic exposure to air pollution may contribute to a state of environmental oxidative priming that modifies the response to cardiac surgery. Through persistent effects on endothelial function, mitochondrial integrity, nitric oxide signaling, inflammatory regulation, and endogenous antioxidant defenses, environmental exposures may shape the biological substrate upon which surgical injury occurs. In this framework, CABG does not act in isolation. Instead, it represents an acute physiological challenge superimposed upon a cardiovascular system already conditioned by decades of environmental influence.

Such a perspective helps reconcile several longstanding observations in cardiac surgery. Patients with apparently similar clinical profiles frequently experience markedly different postoperative trajectories. Some tolerate substantial operative stress with minimal consequences, whereas others develop atrial fibrillation, neurological injury, renal dysfunction, impaired recovery, or premature graft failure despite technically successful procedures. Traditional risk factors undoubtedly contribute to these differences, yet they do not fully explain them. The concept of environmental oxidative priming suggests that part of this variability may reside in biological processes that begin long before surgery becomes necessary.

Importantly, this hypothesis does not imply that air pollution is a dominant determinant of outcome or that oxidative stress represents a universal explanation for postoperative complications. Such conclusions would exceed the available evidence. Rather, the available data support a more nuanced interpretation. Air pollution appears capable of influencing many of the same pathways that govern adaptation to surgical stress, including endothelial homeostasis, mitochondrial resilience, vascular repair, and inflammatory control. Consequently, environmental exposure may alter the threshold at which complications occur without necessarily acting as a direct causal trigger.

The implications extend beyond cardiac surgery. Environmental exposure is increasingly recognized as a contributor to cardiovascular aging itself. Endothelial senescence, mitochondrial dysfunction, vascular stiffening, chronic inflammation, and impaired regenerative capacity are hallmarks of both aging and long-term pollution exposure. The possibility that environmental factors accelerate the biological processes that determine surgical resilience raises important questions regarding how cardiovascular risk is conceptualized and measured. Future risk models may need to account not only for disease burden but also for the environmental conditions under which that disease developed.

At present, however, the field remains in its early stages. Direct evidence linking pollution exposure, oxidative biomarkers, and CABG outcomes within the same patient populations remains limited. Exposure assessment remains imperfect, oxidative stress remains difficult to quantify, and the biological pathways connecting environmental injury to clinical events remain incompletely defined. These limitations should encourage caution but not dismissal. Many of the most important advances in cardiovascular medicine began with observations that were biologically plausible long before they became clinically measurable.

Future investigations integrating individual exposure assessment, redox biomarkers, endothelial phenotyping, mitochondrial function, and long-term clinical outcomes will be essential to determine whether environmental oxidative priming represents a measurable and clinically actionable determinant of perioperative risk.

Perhaps the most important implication of this review is conceptual rather than mechanistic. The long-term success of CABG depends not only on the operation itself but also on the biological environment in which recovery, adaptation, and cardiovascular aging subsequently occur. Air pollution represents one component of that environment—continuous, often invisible, and largely absent from contemporary surgical thinking. Yet it may influence the same molecular pathways that determine resilience to operative stress and durability of surgical benefit.

Whether environmental oxidative priming ultimately proves to be a major determinant of outcome or a modest contributor to existing risk remains to be established. Nevertheless, the hypothesis challenges us to broaden our perspective. It encourages a shift from viewing CABG as an isolated procedural event toward understanding it as one chapter in a lifelong interaction between environmental exposure, biological adaptation, and cardiovascular disease.

In the coming decades, the most significant advances in cardiac surgery may not arise solely from new conduits, novel devices, or refined operative techniques. They may also emerge from a deeper understanding of the biological context in which surgery is performed. Recognizing the role of environmental exposures in shaping cardiovascular resilience represents an important step toward that broader vision and may ultimately help explain why the benefits of surgical revascularization vary so markedly among patients who appear, at least clinically, to be the same.

## Figures and Tables

**Table 1:** Major Air Pollutants Relevant to CABG: Sources, Exposure Characteristics, and Biological Signatures.

Pollutant	Main Sources	Typical Exposure Setting	Principal Oxidative Mechanisms	Cardiovascular Effects	Potential CABG Relevance	References
PM2.5	Traffic, industry, combustion	Urban	Mitochondrial ROS, endothelial injury	Atherosclerosis, thrombosis	POAF, graft dysfunction, MACE	[76,77,78]
PM10	Agriculture, dust, biomass	Rural/mixed	Pulmonary inflammation	Systemic inflammation	AKI, inflammatory complications	[79,80,81]
NO_2_	Traffic emissions	Urban	NO depletion, oxidative stress	Vascular stiffness	Endothelial dysfunction, graft adaptation	[82,83,84]
Ozone (O_3_)	Photochemical pollution	Summer	Oxidative injury	Endothelial dysfunction	Long-term vascular aging	[85,86,87]

Abbreviations: AKI, acute kidney injury; CABG, coronary artery bypass grafting; MACE, major adverse cardiovascular events; NO_2_, nitrogen dioxide; O_3_, ozone; PM2.5, particulate matter with an aerodynamic diameter ≤ 2.5 μm; PM10, particulate matter with an aerodynamic diameter ≤ 10 μm; ROS, reactive oxygen species.

**Table 2:** Shared Molecular Pathways Between Air Pollution and CABG.

Pathway	Air P.	CABG Evidence	Consequence	Clinical Outcome	Pathway	References
Endothelial dysfunction	PM2.5, NO_2_	CPB, I/R injury	Reduced vascular reserve	Stroke, graft failure	Endothelial dysfunction	[124,125,126]
Mitochondrial dysfunction	ROS generation	Reperfusion injury	Energy failure	Myocardial injury	Mitochondrial dysfunction	[127,128]
Nitric oxide depletion	Oxidative scavenging	Endothelial injury	Vasoconstriction	POAF, graft dysfunction	Nitric oxide depletion	[126,129]
NADPH oxidase activation	Pollutant-triggered	Surgical inflammation	ROS amplification	Multi-organ injury	NADPH oxidase activation	[130,131]
Nrf2 impairment	Chronic exposure	I/R injury	Reduced antioxidant defense	Poor recovery	Nrf2 impairment	[127,132]

Abbreviations: CPB—cardiopulmonary bypass, I/R—ischemia–reperfusion, NO_2_—nitrogen dioxide, Nrf2—nuclear factor erythroid 2-related factor 2, PM2.5—particulate matter with an aerodynamic diameter ≤ 2.5 μm.

**Table 3:** Candidate Biomarkers of Environmental Oxidative Priming Before CABG.

Biological Domain	Biomarker	Biological Significance	Evidence	Potential Perioperative Application	References
Oxidative stress	MDA	Lipid peroxidation	Strong	Baseline oxidative burden	[141,142]
	8-OHdG	DNA oxidation	Strong	Oxidative injury	[143]
	F2-isoprostanes	ROS activity	Strong	Oxidative phenotype	[144]
Antioxidant defense	GSH/GSSG	Redox reserve	Moderate	Antioxidant capacity	[145]
	SOD	Enzymatic defense	Moderate	Oxidative resilience	[146]
	Catalase	Antioxidant enzyme	Moderate	Redox balance	[147,148]
Endothelial dysfunction	ADMA	NO inhibition	Strong	Endothelial reserve	[149]
	VCAM-1	Endothelial activation	Strong	Vascular inflammation	[150]
	ICAM-1	Leukocyte adhesion	Strong	Endothelial injury	[151]
Inflammation	hsCRP	Systemic inflammation	Strong	Risk stratification	[152]
	IL-6	Cytokine activation	Strong	Surgical inflammation	[153]
	TNF-α	Chronic inflammation	Moderate	Biological priming	[154,155]
Mitochondrial dysfunction	mtDNA	Mitochondrial injury	Emerging	Reperfusion injury	[156]
	Cytochrome c	Cell death	Emerging	Myocardial injury	[157]

Abbreviations: 8-OHdG, 8-hydroxy-2′-deoxyguanosine; ADMA, asymmetric dimethylarginine; DNA, deoxyribonucleic acid; GSH/GSSG, reduced glutathione/oxidized glutathione ratio; hsCRP, high-sensitivity C-reactive protein; ICAM-1, intercellular adhesion molecule-1; IL-6, interleukin-6; MDA, malondialdehyde; mtDNA, mitochondrial DNA; NO, nitric oxide; ROS, reactive oxygen species; SOD, superoxide dismutase; TNF-α, tumor necrosis factor-α; VCAM-1, vascular cell adhesion molecule-1.

**Table 4:** Potential Modifiers of Susceptibility to Cardiopulmonary Bypass-Induced Oxidative Stress.

Patient Characteristic	Baseline Oxidative Phenotype	Potential Interaction with CPB	Predicted Impact of Environmental Oxidative Priming	Hypothesized Benefit from Reduced Oxidative Burden (Off-Pump CABG)	References
High long-term PM2.5 exposure	Endothelial dysfunction, reduced antioxidant reserve	Exaggerated inflammatory and oxidative response	High	High	[158,159]
Diabetes mellitus	Chronic ROS generation, NO depletion	Amplified reperfusion injury	High	High	[160,161,162,163]
Chronic kidney disease	Impaired redox buffering capacity	Greater susceptibility to microvascular injury	High	High	[164,165]
Frailty	Reduced physiological resilience	Lower threshold for organ dysfunction	High	High	[166,167]
Advanced age	Endothelial senescence, mitochondrial dysfunction	Reduced tolerance to oxidative stress	Moderate–High	Moderate–High	[168,169]
COPD	Pulmonary and systemic inflammation	Enhanced inflammatory activation	Moderate–High	Moderate	[170,171]
Obesity/metabolic syndrome	Chronic inflammatory activation	Increased oxidative burden	Moderate–High	Moderate	[172,173,174]
Low pollution exposure and preserved physiological reserve	Intact compensatory mechanisms	Greater tolerance of oxidative stress	Low	Uncertain	-----

Abbreviations: CABG—coronary artery bypass grafting, COPD—chronic obstructive pulmonary disease, CPB—cardiopulmonary bypass, NO—nitric oxide, PM2.5—particulate matter with an aerodynamic diameter ≤ 2.5 μm, ROS—reactive oxygen species.

**Table 5:** Evidence Linking Air Pollution to Outcomes Relevant for CABG.

Outcome	Evidence in General Population	Proposed CABG Relevance	Strength of Evidence	References
Atrial fibrillation	Strong	High	Strong	[175,176]
Stroke	Strong	Moderate–High	Moderate	[177,178]
AKI	Moderate	Moderate	Emerging	[179,180]
Heart failure	Strong	High	Strong	[181]
Mortality	Strong	High	Strong	[182]
Graft failure	Limited	Hypothesized	Weak	[183]

Abbreviations: AKI–acute kidney injury, CABG—coronary artery bypass grafting.

**Table 6:** Early vs. Late Outcomes After CABG Potentially Influenced by Environmental Oxidative Priming.

Time Horizon	Outcome	Mechanism	Pollution Contribution	References
Days	POAF	Oxidative atrial remodeling	High	[175,189]
Days	Stroke	Cerebrovascular vulnerability	Moderate	[178,190,191]
Days	AKI	Microvascular injury	Moderate	[192,193,194]
Months	Graft adaptation	Endothelial dysfunction	Moderate	[195]
Years	MACE	Persistent vascular aging	High	[183,196]
Years	Mortality	Cumulative oxidative burden	High	[197]

Abbreviations: AKI—acute kidney injury, CABG—coronary artery bypass grafting, MACE—major adverse cardiovascular events, POAF—postoperative atrial fibrillation.

**Table 7:** Biomarkers for Future CABG-Air Pollution Research.

Biological Domain	Representative Biomarkers	Biological Significance	Potential CABG Application	References (Related to CAD)
Oxidative stress	MDA, 8-OHdG, oxLDL	Oxidative injury	Baseline oxidative burden	[210,211]
Endothelial dysfunction	ADMA, VCAM-1, ICAM-1, NO metabolites	Endothelial injury	Graft adaptation	[212,213,214]
Inflammation	hsCRP, IL-6, TNF-α, MPO	Immune activation	POAF, AKI	[215,216]
Mitochondrial dysfunction	mtDNA, cytochrome c, lactate	Energetic failure	Ischemia-reperfusion susceptibility	[217,218]

Abbreviations: 8-OHdG, 8-hydroxy-2′-deoxyguanosine; ADMA, asymmetric dimethylarginine; AKI, acute kidney injury; CAD—coronary artery bypass grafting, CABG, coronary artery bypass grafting; hsCRP, high-sensitivity C-reactive protein; ICAM-1, intercellular adhesion molecule-1; IL-6, interleukin-6; MDA, malondialdehyde; MPO, myeloperoxidase; mtDNA, mitochondrial DNA; NO, nitric oxide; oxLDL, oxidized low-density lipoprotein; POAF, postoperative atrial fibrillation; TNF-α, tumor necrosis factor-α; VCAM-1, vascular cell adhesion molecule-1.

**Table 8:** Research Gaps and Future Directions.

Knowledge Gap	Current Evidence	Limitation	Proposed Study Design
Pollution and POAF	Indirect	No CABG cohorts	Prospective multicenter cohort
Pollution and graft patency	Minimal	No longitudinal data	CTA/angiographic follow-up
On-pump vs. off-pump interactions	Theoretical	No exposure stratification	Exposure-adjusted trials
Biomarker validation	Preliminary	Small studies	Mechanistic translational studies
Environmental risk models	None	Not integrated into EuroSCORE/STS	Predictive modeling studies

Abbreviations: CABG, coronary artery bypass grafting.

**Table 9:** Potential Clinical Implications of Environmental Oxidative Priming in CABG.

Stage of Care	Potential Application
Preoperative assessment	Incorporation of exposure metrics
Risk stratification	Environmental risk modifiers
Procedure selection	On-pump vs. off-pump consideration
Perioperative management	Antioxidant/anti-inflammatory strategies
Follow-up	Geographic exposure monitoring
Research	Integration with exposome-based models

## Data Availability

No new data were created in the process of manuscript writing.

## Abbreviations

The following abbreviations are used in this manuscript:

AKI	Acute kidney injury
ATP	Adenosine triphosphate
CABG	Coronary artery bypass grafting
CKD	Chronic kidney disease
COPD	Chronic obstructive pulmonary disease
CPB	Cardiopulmonary bypass
DNA	Deoxyribonucleic acid
EuroSCORE II	European System for Cardiac Operative Risk Evaluation II
IL	Interleukin
I/R	Ischemia–reperfusion
LDL	Low-density lipoprotein
MACE	Major adverse cardiovascular events
MPO	Myeloperoxidase
NADPH	Nicotinamide adenine dinucleotide phosphate
NETs	Neutrophil extracellular traps
NLRP3	NOD-, LRR- and pyrin domain-containing protein 3
NO	Nitric oxide
NO_2_	Nitrogen dioxide
NOx	Nitrogen oxides
Nrf2	Nuclear factor erythroid 2–related factor 2
O_3_	Ozone
PM	Particulate matter
PM2.5	Particulate matter with an aerodynamic diameter ≤ 2.5 μm
PM10	Particulate matter with an aerodynamic diameter ≤ 10 μm
POAF	Postoperative atrial fibrillation
ROS	Reactive oxygen species
STS	Society of Thoracic Surgeons
